# Supplementary material for: Trajectories of self-rated health in an older general population and their determinants: the Lifelines Cohort Study
Source: BMJ Open. 2020 Feb 18;10(2):e035012. doi: 10.1136/bmjopen-2019-035012 (PMC7045095; doi:10.1136/bmjopen-2019-035012)
Supplement: Supplementary data [file bmjopen-2019-035012supp001.pdf]

APPENDIX A: PHYSIOLOGICAL MARKER SPECIFICATION

Table A1: Cut-offs used to define normal and affected values for markers per organsystem.

**Lung function**		
FEV1 (L) /FVC (L) ratio (multiplied by 100%)	Cut-off scores used (Quanjer et al., 2012):<br>Normal: ≥ 70%<br>Affected < 70%	
**Renal function**		
eGFR (in ml/min/1.73m2)	Estimated with the Cockcroft Gauld formula using serum creatine in umol/l (adjusted for age, sex, weight) (Cockcroft & Gault, 1976).<br>Cut-off scores used (Traynor, Mactier, Geddes, & Fox, 2006):<br>Normal: ≥90 ml/min/1.73m^2<br>Affected: <90 ml/min/1.73m^2	
**Endocrine function**[1]		
TSH (mIU/L)	Normscores (lab standards UMCG):<br>Low: <0.5 mIU/L<br>Normal: 0.5 – 4.0 mIU/L<br>High: ≥ 4.0 mIU/L	
fT4 (pmol/L)	Normscores (Boesten et al., 2012):<br>Low: < 11.0 pmol/L<br>Normal: 11.0 – 19.5 pmol/L<br>High: > 19.5 pmol/L	
**Immune function**		
Hb (mmol/L)	Different cut-offs used for men and women (lab standards UMCG).<br>Cut off used:<br>Men:<br>Normal: ≥8.5 and ≤ 11 mmol/L<br>Affected: <8.5, >11 mmol/L	 <br>Women:<br>Normal: ≥7.5 and ≤ 10 mmol/L<br>Affected: <7.5, >10 mmol/L
**Liver function**		
Hepatic Steatosis Index	Cut off used (Lee et al., 2010; Meems et al., 2015):<br>Normal: ≤ 36<br>Affected >36	
**Cognitive function**		
MMSE	Adjusted for level of education: primary education or less (max.6 years) and secondary or higher (>6 years) (Schmand, Lindeboom, Hooijer, & Jonker, 1995).<br>Cut-off used:<br>≤ Primary:<br>Normal: ≥25<br>Affected: <25	 <br><br>≥ Secondary:<br>Normal ≥27<br>Affected: <27
**Body composition**		
BMI (for Caucasian)	Age adjusted BMI cutoffs were used (Winter, Macinnis, Wattanapenpaiboon, & Nowson, 2014).<br>Cut offs used:<br>Normal: ≥23.0 BMI <30<br>Affected: <23 & BMI ≥30	
**Cardiovascular function**[2]		

Feenstra M, *et al. BMJ Open* 2020; 10:e035012. doi: 10.1136/bmjopen-2019-035012

SBP (mmHg)	Adjusted for age ((European Society of Hypertension/ European Society of Cardiology, 2014))<br>Cut-offs used:<br>Aged <80: Normal: ≤ 140 mmHg; High: >140 mmHg<br>Aged ≥80: Normal: ≤ 160 mmHg; High: >160 mmHg
DBP (mmHg)	Cut-off used (European Society of Hypertension/ European Society of Cardiology, 2014):<br>Normal: <90 mmHg<br>High: ≥90 mmHg
Total cholesterol (mmol/L)/ HDL (mmol/L) ratio	Cut offs used (European Society of Cardiology / European Atherosclerosis Society, 2016; Landelijke werkgroep Cardiovasculair risicomanagement, 2012):<br>Normal: <5.0<br>High ≥ 5.0
**Glucose metabolism**	
HbA1c (mmol (HbA1c) / mol (Hb))	Cut offs used (Fried et al., 2009):<br>Normal: < 48 mmol/mol (corresponding to 6,5% of total Hb)<br>Affected: ≥ 48 mmol/mol

Abbreviations: FER, forced expiratory ratio; eGFR, estimated glomerular filtration rate; TSH, thyroid stimulating hormone; FSH, follicle-stimulating hormone; HB, hemoglobin; MMSE, mini mental state examination; BMI, body mass index; SBP, systolic blood pressure; DBP, diastolic blood pressure; HDL, high density lipoprotein; HbA1C, glycated hemoglobin.

1. TSH cut-offs were interpreted with fT4; both TSH and fT4 should be in the normal range to score 'normal' concerning the endocrine system.

2. Blood pressure was interpreted with cholesterol levels; both diastolic and systolic bloodpressure and cholesterol/HDL ratio or should in the normal range to score 'normal' concerning the cardiovascular system.

References:

Boesten, L. S. M., Brugts, M. P., & van Rossum, A. P. (2012). Schildklierdiagnostiek : discrepantie FT4 en TSH. *Nederlands Tijdschrift Voor Geneeskunde*, *156*, A4167V.

Cockcroft, D. W., & Gault, H. (1976). Prediction of Creatinine Clearance from Serum Creatinine. *Nephron*, *16*(1), 31–41. European Society of Cardiology / European Atherosclerosis Society. (2016). 2016 ESC/EAS guidelines for the management of dyslipidemias. *European Heart Journal*, *37*, 2999–3058.

European Society of Hypertension/ European Society of Cardiology. (2014). 2014 guidelines for the management of arterial hypertension. *Primary Care Cardiovascular Journal*, *7*(2), 85–88.

Fried, L. P., Xue, Q. L., Cappola, A. R., Ferrucci, L., Chaves, P., Varadhan, R., … Bandeen-Roche, K. (2009). Nonlinear multisystem physiological dysregulation associated with frailty in older women: Implications for etiology and treatment. *Journals of Gerontology - Series A Biological Sciences and Medical Sciences*, *64*(10), 1049–1057.

Landelijke werkgroep Cardiovasculair risicomanagement. (2012). Dutch standard of cardiovasculair riskmanagement. *Huisarts Wet*, *55*(1), 14–28.

Lee, J., Kim, D., Jung, H., Lee, C., In, J., Kim, W., … Lee, H. (2010). Hepatic steatosis index : A simple screening tool reflecting nonalcoholic fatty liver disease. *Digestive and Liver Disease*,

42(7), 504–509.

Meems, L. M. G., De Borst, M. H., Postma, D. S., Vonk, J. M., Kremer, H. P. H., Schuttelaar, M. L. A., … De Boer, R. A. (2015). Low levels of Vitamin D are associated with multimorbidity: Results from the LifeLines Cohort Study. *Annals of Medicine*, *47*(6), 474–481.

Quanjer, P. H., Stanojevic, S., Cole, T. J., Baur, X., Hall, G. L., Culver, B. H., … Schindler, C. (2012). Multi-ethnic reference values for spirometry for the 3-95-yr age range: The global lung function 2012 equations. *European Respiratory Journal*, *40*(6), 1324–1343.

Schmand, B., Lindeboom, J., Hooijer, C., & Jonker, C. (1995). Relation between education and dementia : the role of test bias revisited. *Journal of Neurology, Neurosurgery and Psychiatry*, *59*, 170–174.

Traynor, J., Mactier, R., Geddes, C. C., & Fox, J. G. (2006). How to measure renal function in clinical practice: Estimated glomerular filtration rate in general practice. *Bmj*, *333*(7574), 918.2.

Winter, J. E., Macinnis, R. J., Wattanapenpaiboon, N., & Nowson, C. A. (2014). BMI and all-cause mortality in older adults: a meta-analysis. *Am J Clin Nutr*, *99*, 875–890.

APPENDIX B: FLOWCHART OF STUDY SAMPLE

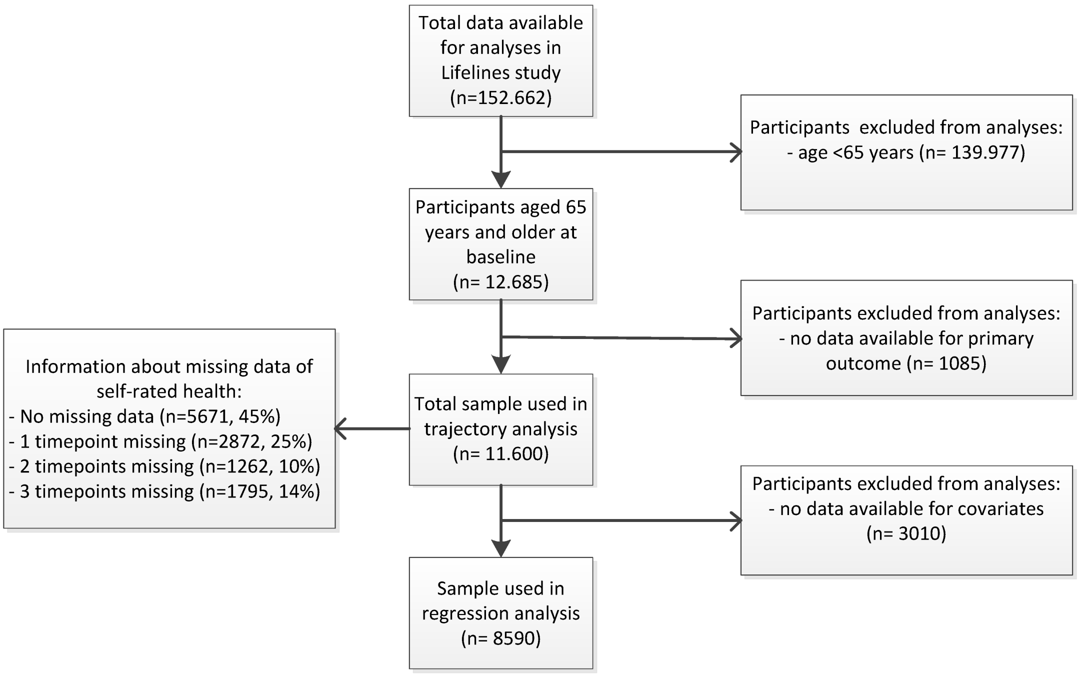

Figure B1. Flow of selection of study sample.

Feenstra M, *et al. BMJ Open* 2020; 10:e035012. doi: 10.1136/bmjopen-2019-035012

APPENDIX C: BASIC MODEL SPECIFICATIONS

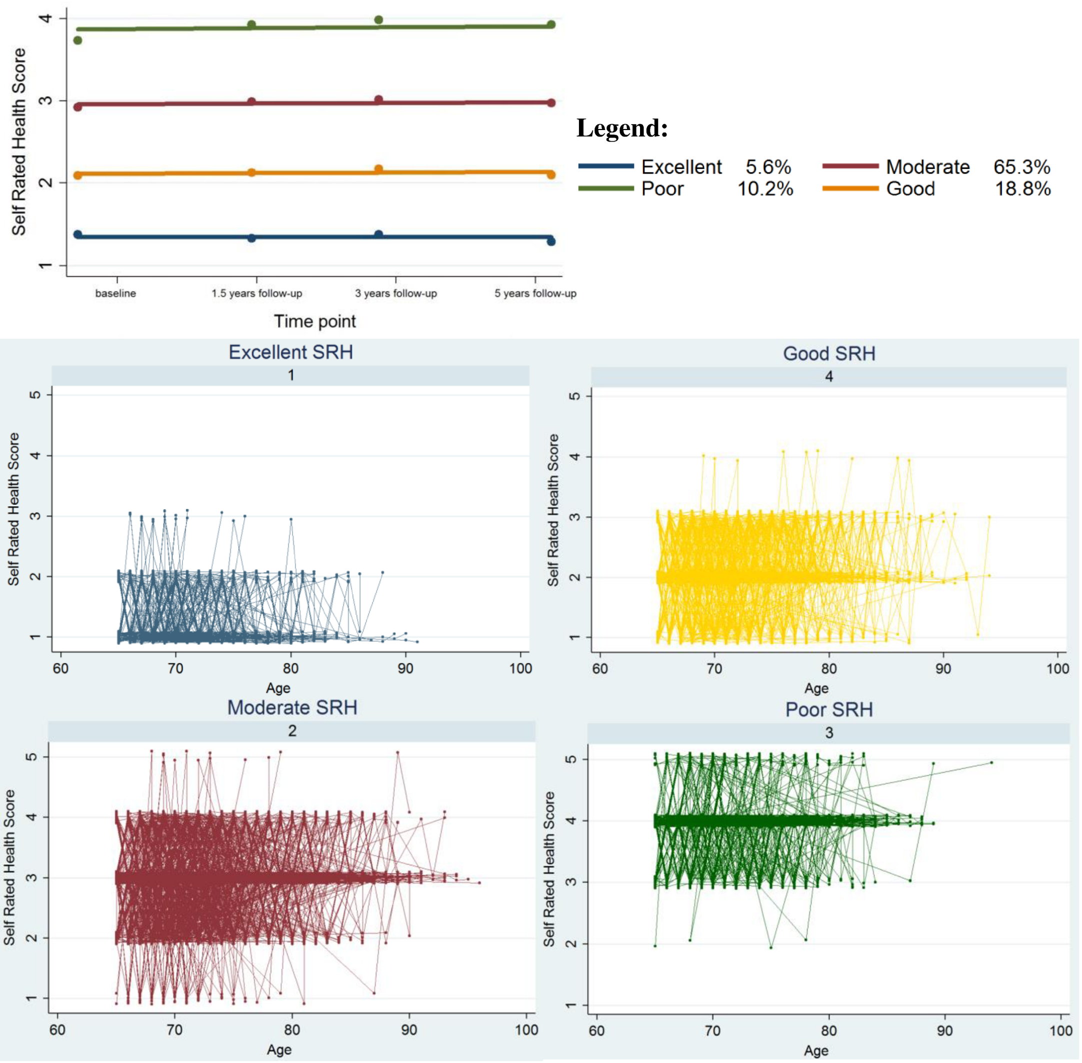

Figure C1: Basic mean trajectory groups of SRH (a), and observed individual trajectories per trajectory group (b-e) over five years of 11.600 people aged 65 years and older of the Lifelines Cohort.
a. Dots represent the mean observed value per measurement moment; solid lines represent fit lines; dotted lines represent 95% confidence intervals of the fit lines.
b-e. Jittering was used for adding random noise to make all individual scores integer to avoid overlap of individual trajectories for people with identical trajectories.

Feenstra M, *et al. BMJ Open* 2020; 10:e035012. doi: 10.1136/bmjopen-2019-035012

Table C1: Bayesian Information Criteria and probability estimation Jeffreys's scale of evidence for Bayes factors of crude trajectory calculations with fixed quadratic growth terms used to select adequate number of groups.

**No. of groups**	**BIC** (n=11.600)	**Probability correct model**
2	-35016.79	0
3	-33001.46	0
4	-32240.77	0.98
5	-32259.49	< 0.01
6	-32278.20	< 0.01
7	-32244.54	0.02

Table C2: Posterior diagnostics of model performance of basic trajectory model.

**Group**	**Model estimate** ***(π^)***	**95% CI**	**Proportion classified** ***(p^)***	**Ave. PP**	**Odds correct classification**
1	.056	(.050; .062)	.052	.892	138.3
2	.653	(.642; .664)	.662	.941	8.4
3	.102	(.095; .109)	.104	.852	50.7
4	.188	(.179; .198)	.182	.863	27.1

Feenstra M, *et al. BMJ Open* 2020; 10:e035012. doi: 10.1136/bmjopen-2019-035012

APPENDIX D: SENSITIVITY ANALYSES

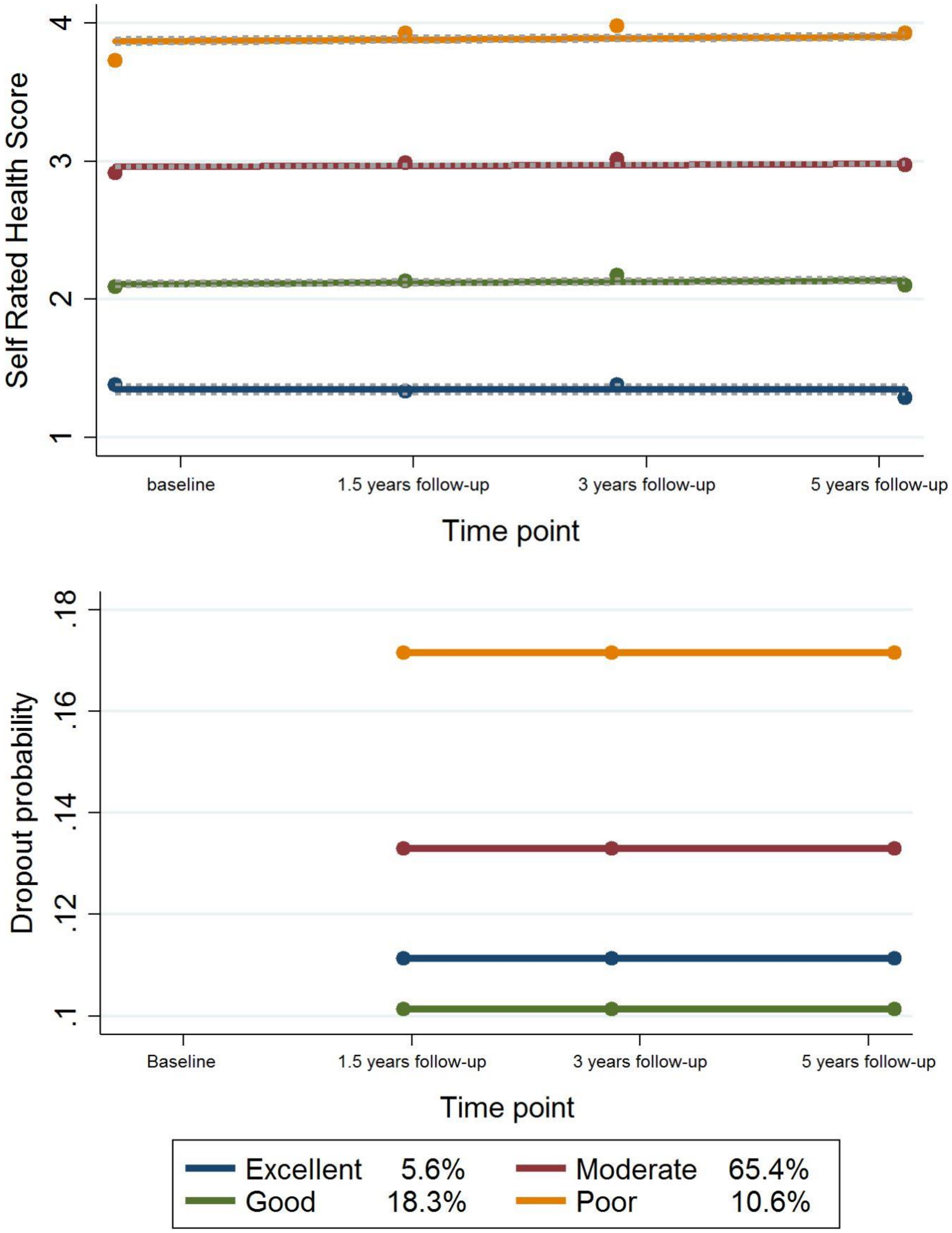

Figure D1: Trajectories of SRH jointly modelled with attrition. The upper plot represent trajectories of SRH accounted for attrition risk with probability for dropout per trajectory is presented in the lower plot. Dots represent the mean observed value per measurement moment; solid lines represent fit lines; dotted lines in the upper plot represent 95% confidence intervals of the fit lines.

Feenstra M, *et al. BMJ Open* 2020; 10:e035012. doi: 10.1136/bmjopen-2019-035012

Table D1. Comparison of posterior probability of assignment for the basic model, the model including covariates / risk factors, and the trajectory model that jointly modelled attrition (sensitivity analysis).

Group Allocation	N (%)	Excellent	Good	Moderate	Poor
**Basic model (step 1): posterior probability of assignment**					
Excellent	607 (5.6)	**0.89**	<0.01	0.01	0.05
Good	2111 (18.8)	0.11	**0.86**	0.06	<0.01
Moderate	8762 (65.3)	<0.01	0.15	**0.91**	0.08
Poor	1205 (10.2)	<0.01	<0.01	0.03	**0.85**
**Model with covariates (step 3): posterior probability of assignment**					
Excellent	471 (5.5)	**0.91**	0.05	<0.01	<0.01
Good	1716 (20.0)	0.09	**0.87**	0.04	<0.01
Moderate	5637 (65.6)	<0.01	0.08	**0.95**	0.09
Poor	766 (8.9)	<0.01	<0.01	0.02	**0.91**
**Model with attrition (sensitivity analysis): posterior probability of assignment**					
Excellent	609 (5.7)	**0.90**	0.05	0.01	<0.01
Good	2123 (18.7)	0.10	**0.86**	0.05	<0.01
Moderate	8762 (65.3)	<0.01	0.09	**0.91**	0.14
Poor	1191 (10.3)	<0.01	<0.01	0.04	**0.86**

*Rows may add to more than 1.0 due to rounding.

Feenstra M, *et al. BMJ Open* 2020; 10:e035012. doi: 10.1136/bmjopen-2019-035012
