# Supplementary material for: Trajectories of self-rated health in an older general population and their determinants: the Lifelines Cohort Study
Source: BMJ Open. 2020 Feb 18;10(2):e035012. doi: 10.1136/bmjopen-2019-035012 (PMC7045095; doi:10.1136/bmjopen-2019-035012)
Supplement: Author's manuscript [file bmjopen-2019-035012.draft_revisions.pdf]

BMJ Open is committed to open peer review. As part of this commitment we make the peer review history of every article we publish publicly available.

When an article is published we post the peer reviewers' comments and the authors' responses online. We also post the versions of the paper that were used during peer review. These are the versions that the peer review comments apply to.

The versions of the paper that follow are the versions that were submitted during the peer review process. They are not the versions of record or the final published versions. They should not be cited or distributed as the published version of this manuscript.

BMJ Open is an open access journal and the full, final, typeset and author-corrected version of record of the manuscript is available on our site with no access controls, subscription charges or pay-per-view fees (http://bmjopen.bmj.com).

If you have any questions on BMJ Open's open peer review process please email info.bmjopen@bmj.com

Trajectories of self-rated health in an older general population and their determinants: The Lifelines Cohort Study

Journal:	*BMJ Open*
Manuscript ID	bmjopen-2019-035012
Article Type:	Original research
Date Submitted by the Author:	17-Oct-2019
Complete List of Authors:	Feenstra, Marlies; University of Groningen; Universitair Medisch Centrum Groningen, Internal Medicine and Geriatrics<br>van Munster, Barbara; Universitair Medisch Centrum Groningen, Internal Medicine and Geriatrics; Gelre Ziekenhuizen, Geriatrics<br>Macneil-Vroomen , Janet; Amsterdam Universitair Medische Centra, Geriatrics ; Yale School of Medicine<br>De Rooij, Sophia; Universitair Medisch Centrum Groningen, Internal Medicine and Geriatrics; Medical Centre Twente<br>Smidt, Nynke; Universitair Medisch Centrum Groningen, Epidemiology; Universitair Medisch Centrum Groningen, Internal Medicine and Geriatrics
Keywords:	EPIDEMIOLOGY, PUBLIC HEALTH, Risk management < HEALTH SERVICES ADMINISTRATION & MANAGEMENT

SCHOLARONE™
Manuscripts

I, the Submitting Author has the right to grant and does grant on behalf of all authors of the Work (as defined in the below author licence), an exclusive licence and/or a non-exclusive licence for contributions from authors who are: i) UK Crown employees; ii) where BMJ has agreed a CC-BY licence shall apply, and/or iii) in accordance with the terms applicable for US Federal Government officers or employees acting as part of their official duties; on a worldwide, perpetual, irrevocable, royalty-free basis to BMJ Publishing Group Ltd ("BMJ") its licensees and where the relevant Journal is co-owned by BMJ to the co-owners of the Journal, to publish the Work in this journal and any other BMJ products and to exploit all rights, as set out in our licence.

The Submitting Author accepts and understands that any supply made under these terms is made by BMJ to the Submitting Author unless you are acting as an employee on behalf of your employer or a postgraduate student of an affiliated institution which is paying any applicable article publishing charge ("APC") for Open Access articles. Where the Submitting Author wishes to make the Work available on an Open Access basis (and intends to pay the relevant APC), the terms of reuse of such Open Access shall be governed by a Creative Commons licence – details of these licences and which Creative Commons licence will apply to this Work are set out in our licence referred to above.

Other than as permitted in any relevant BMJ Author's Self Archiving Policies, I confirm this Work has not been accepted for publication elsewhere, is not being considered for publication elsewhere and does not duplicate material already published. I confirm all authors consent to publication of this Work and authorise the granting of this licence.

For peer review only

Trajectories of self-rated health in an older general population and their determinants: The Lifelines Cohort Study

Marlies Feenstra[a*], Barbara C van Munster[a,b], Janet L MacNeil Vroomen[c,d], Sophia E de Rooij[a,e], Nynke Smidt[a,f]

[a] University of Groningen, University Medical Center Groningen, Department of Internal Medicine and Geriatrics, Groningen, The Netherlands

[b] Gelre Hospitals, Department of Geriatrics, Apeldoorn, The Netherlands

[c] Amsterdam University Medical Centers, Department of Internal Medicine, Section of Geriatrics, Amsterdam, The Netherlands

[d] Yale School of Medicine, Department of Internal Medicine, Section of Geriatrics, New Haven, The United States of America

[e] Medical School Twente, Medical Spectrum Twente, Enschede, The Netherlands

[f] University of Groningen, University Medical Center Groningen, Department of Epidemiology, Groningen, The Netherlands

[*]**Corresponding author**: Marlies Feenstra

University of Groningen, University Medical Center Groningen

Department of Internal Medicine and Geriatrics

PO Box 30001, 9700 RB Groningen, The Netherlands

Tel: (+31) 50 361 3219 E-mail: m.feenstra01@umcg.nl

For peer review only

For peer review only - http://bmjopen.bmj.com/site/about/guidelines.xhtml

ABSTRACT

Objectives:

Poor self-rated health (SRH) is a strong predictor of premature mortality in older adults. Trajectories of poor SRH are associated with multimorbidity and unhealthy behaviors. Whether trajectories of SRH are associated with deviating biomarkers is unclear. This study identified trajectories of self-rated health (SRH) and investigated the associations of trajectory membership with chronic diseases, health risk behaviors, and biomarkers in community-dwelling older adults.

Study design and setting:

Prospective general population cohort

Participants:

Trajectories of SRH over 5 years were identified using data of 11 600 participants aged 65 years and older of the Lifelines Cohort Study.

Outcome measures:

Trajectories of SRH were the main outcome. Covariates included demographics (age, gender, education), chronic diseases, health-risk behavior (physical activity, smoking, drinking), and biomarkers (BMI, cardiovascular function , lung function , glucose metabolism , hematological condition , endocrine function, renal function, liver function, and cognitive function).

Results:

Four stable trajectories were identified, including excellent (n = 607, 6%), good (n = 2111, 19%), moderate (n = 7677, 65%), and poor SRH (n = 1205, 10%). Being female (OR: 1.5; 95%CI: 1.1 - 2.0), low education (OR: 2.1; 95%CI: 1.4 - 2.9), one (OR: 10.4; 95%CI: 7.5 - 14.7) or multiple chronic diseases (OR: 37.7; 95%CI: 22.5 -72.3), smoking (OR: 1.9; 95% CI:

For peer review only - http://bmjopen.bmj.com/site/about/guidelines.xhtml

1.0 - 3.4), physical inactivity (OR: 3.1; 95%CI: 1.8 - 5.3), alcohol abstinence (OR: 2.4; 95%CI: 1.5 - 3.8), and deviating biomarkers (OR: 1.5; 95%CI: 1.0 - 2.0) increase the odds for poor SRH trajectory membership compared to excellent SRH trajectory membership.

Conclusion:

SRH of community-dwelling older adults is stable over time with the majority (65%) having moderate SRH. Older adults reporting poor SRH often have unfavorable health status.

Key words:

Longitudinal; Trajectory; Aging; Biomarkers; Health risk behavior; Multimorbidity.

STRENGHTS AND LIMITATIONS OF THIS STUDY

- This study concerns the evaluation of biomarkers as a determinant of self-rated health trajectories.
- The study results are representative for Dutch community dwelling adults aged 65 years and older.
- Reverse causation could not be eliminated.
- The number of chronic conditions were based on self-report, this could have caused non-differential misclassification bias.

Word count:

Abstract: 279

Main text: 3200

Tables: 4

Figures: 1

Appendices: 4 (A - D)

For peer review only - http://bmjopen.bmj.com/site/about/guidelines.xhtml

BACKGROUND

Self-rated health (SRH) is often used as a measure of global health and as a supplement to more objective clinical measures of physical health, such as presence of disease and disability (1,2). In older adults, poor SRH is an independent and strong predictor of premature mortality (2,3). However, evidence for factors associated with poor SHR are predominantly cross-sectional and longitudinal evidence is required. Analysis of latent clusters of individuals who follow a similar pattern of SRH over time, so called trajectory analysis, can be used to explore the course of SRH in time within a certain population (4). Few studies have studied SRH in community-dwelling older adults by trajectory analysis revealing various numbers of identified trajectories (5–7). Distinct trajectories of SRH varied from persistently good (5,6), persistently moderate (6,7), persistently poor (5,6), declining (5–7) to improving trajectories of SRH (5). People in declining SRH trajectories were differentiated at baseline by older age, lower education level, and an increased number of chronic conditions compared to people in consistently good SRH trajectories (5–7). However, in these studies, other measures of determinants of health status, such as abnormalities in biomarkers, like blood pressure, thyroid hormone levels, and glycated hemoglobin were not evaluated. Such biomarkers reflect cross-sectional clinical parameters of physiological processes (8). Abnormal physiological processes may indicate pre-clinical prodromal phases of underlying diseases which are suggested to play a role in burden of disease expressed by poor SRH evaluations in older adults (1,3,9,10). We hypothesize that multi-morbidity, health risk behaviors, and deviations in biomarkers are associated with trajectories that lead to poor SRH.

The aim of this study is to identify classes of self-rated health over five years in community-dwelling older adults and to investigate whether group membership of SRH trajectories is associated with self-reported chronic diseases, health risk behaviors, and biomarkers.

For peer review only

For peer review only - http://bmjopen.bmj.com/site/about/guidelines.xhtml

METHODS

Study population

A subsample of the adult Lifelines Cohort Study was used, including participants aged 65 years or older at baseline (n = 12 685) of which data at baseline and three follow-up measurements over five years period were available. A detailed description of the complete Lifelines cohort profile is described elsewhere (11).

Measurements

Primary outcome measure

Self-rated health was assessed at baseline, 1.5 years, 3 years, and 5 years after baseline measurement by means of a self-reported question 'how would you rate your health in general? (excellent, very good, good, fair, poor)' (12,13).

Covariates

Demographics included *age, sex*, and *education level* (low, less than primary through lower secondary; intermediate, upper secondary through post-secondary, non-tertiary; high, short cycle tertiary and higher (14,15)).

Chronic diseases were categorized (none, one, two or more) based on a participant's baseline report on presence of the most burdensome chronic diseases as forecasted for the next decades by (RIVM, 2017), including dementia, myocardial infarction, osteoarthritis, cerebrovascular accident (CVA), diabetes, chronic obstructive pulmonary disease (COPD), cancer, anxiety, and mood disorders.

Health risk behaviors included *physical activity* (≥5, 2-4, ≤2 days/week physically active for at least 30 minutes (16)), *smoking* (never, former, current smoker), *alcohol consumption* (abstainer, low risk, at risk (17)). Low risk drinking is defined as no more than three and four drinks per day for women and men respectively, and no more than seven drinks per week.

For peer review only - http://bmjopen.bmj.com/site/about/guidelines.xhtml

Biomarkers included: body mass index (BMI) as a marker of body composition (18,19); Systolic and diastolic blood pressure was interpreted with total cholesterol and high density lipoprotein (HDL) ratio as a marker of cardiovascular function (18); Forced expired volume in one second (FEV1) and the forced vital capacity (FVC) ratio was used as a marker of lung function (20,21); Glycated hemoglobin (HbA1c) as a marker of glucose metabolism (18,22); Total hemoglobin as a marker of hematological condition (22), Thyroid Stimulating Hormone (TSH) and free thyroxine (fT4) were used as markers of endocrine function (23–25); Estimated glomerular filtration rate (eGFR) by using the Cockcroft Gauld formula was used as a marker of renal function (26–28); Hepatic Steatosis Index (HSI) was used as a marker of liver function (29,30); and the mini mental state examination score (MMSE) was used as a marker of cognitive function (18,31). A detailed description of biomarkers used and clinical cut-offs are presented in Appendix A Table A1. Based on clinical cut-offs, both *individual biomarkers* (normal, abnormal values) and a *sum score of abnormal biomarkers* were used in the analyses (<3 vs. ≥3 abnormal biomarkers).

Statistical analyses

Baseline characteristics of all participants and classified by SRH trajectory groups were expressed in median and interquartile range (IQR) for continuous variables and proportions and percentages for categorical variables. To identify distinct trajectories of self-rated health, latent class analysis were performed by using Group Based Trajectory Modeling (GBTM) (32). The trajectory model was built by a stepwise approach:

Step 1, basic trajectories of SRH: crude trajectories were plotted by using a censored normal model with fixed quadratic growth terms. Two up to six trajectories were considered. The optimal model was selected using Bayesian Information Criterion (BIC), and Bayes factor. BIC is a measure of model fit with higher values indicating better model fit (33). In addition, for all models with varying numbers of groups a BIC based probability estimation

For peer review only - http://bmjopen.bmj.com/site/about/guidelines.xhtml

was calculated using Jeffreys's scale of evidence for Bayes factor (34). After the optimal number of groups was determined, higher (cubic) or lower (linear, constant) order growth terms were added to determine optimal trajectory shape. Optimal shape was determined based on posterior diagnostic criteria. These criteria reflect 1. the probability of a person belonging to the selected trajectory (>0.7), 2. Odds of correct classification (>5.0), 3. close correspondence between the estimate of group membership probability and the proportion of individuals classified to the group (no formal criteria for maximum deviation), and 4. reasonable narrow confidence intervals for the estimates of group membership probability (no formal criteria for maximum deviation) (35).

Step 2, identification of covariates of trajectory membership probability: To estimate associations of cumulative disease burden and trajectories of SRH identified in step 1 multivariable multinomial logistic regression analysis were performed, using the excellent SRH trajectory as reference. Three theoretical models were investigated. *Model 1*: chronic diseases and health behaviors; *Model 2*: model 1 plus biomarkers; *Model 3*: model 1 plus the sum score of abnormal biomarkers. For all determinants, multicollinearity was checked using Pearson's correlations. All models were adjusted for baseline demographic covariates age, sex, and level of education. Model selection was based on lowest BIC, and Akaike's Information Criterion (AIC)(36).

Step 3, the final model adjusted for associated covariates: covariates of the selected model out of step 2 were jointly estimated with the trajectories in step 1. Adding these covariates as risk factors to the model allows to evaluate the influence of one covariate on the probability of belonging to each trajectory taking into account the uncertainty of posterior group membership probability that is introduced by trajectory analysis. Wald statistics were applied for testing the differences between risk factors across trajectory groups.

For peer review only - http://bmjopen.bmj.com/site/about/guidelines.xhtml

For peer review only

Data of participants with missing data of were not imputed (n=1085 (9%)) and were therefore excluded from data analyses. Participants with missing data of the main outcome at three or less time points were imputed using maximum likelihood estimation. The flow of participants from the initial to the analytic sample is presented in Appendix B Figure B1. The data of the 3010 (26%) participants who had missing data for baseline covariates were not imputed. Sensitivity analyses were performed by: 1) rerunning basic trajectory analysis accounting for non-random attrition (dual trajectory modeling), and 2) using a composite score for chronic diseases without anxiety and mood disorders. For all analyses Stata Statistical Software release 14 was used (StataCorp. 2015. College Station, Texas, USA) with the Traj plug-in (37,38).

RESULTS

Study population characteristics

Of all 11 600 participants, median age at baseline was 69 years (range 65 to 93), and 47% were male. Of this sample, 34% reported one chronic disease at baseline, 13% reported multimorbidity (≥2 chronic diseases), 57% had one or two abnormal biomarkers, and 38% had three or more abnormal biomarkers (Table 1). Over five years of follow-up, 497 people died (4%), and 3721 (32%) were lost to follow-up. Their missing data for SRH was imputed for the analysis in step 1. The 3010 (26%) participants who were excluded from the analysis in step 2 and 3 due to missing covariates measured at baseline were older, more often female, lower educated, and had relatively less self-reported chronic diseases, but more abnormal values of biomarkers compared to the participants retained in the analysis (completers) (Table 2). One of the reasons for these missing data was that participant with low cognitive abilities (mini mental state examination <26) had a shorter proxy interview, which was the case in 1261 (42%) of the excluded participants.

For peer review only - http://bmjopen.bmj.com/site/about/guidelines.xhtml

Table 1. Baseline characteristics of all participants aged 65 years and older and categorized by SRH trajectory group.

**Characteristic**	**All**	**1. Excellent**	**2. Good**	**3. Moderate**	**4. Poor**
**n**	**11 600**	**602**	**2111**	**7677**	**1205**
**Demographics**					
Age, median (IQR25;75)	69 (66; 73)	68 (66; 72)	69 (66; 72)	69 (66; 72)	70 (67; 74)
range (years)	65-95	65-90	65-92	65-93	65-90
Sex, n (%) male	5484 (47)	344 (57)	1161 (55)	3523 (46)	456 (38)
Highest level of education, n(%)					
low	6563 (57)	301 (50)	1006 (48)	4482 (58)	774 (64)
intermediate	2037 (18)	107 (18)	407 (19)	1345 (18)	178 (15)
high	2239 (19)	168 (28)	592 (28)	1319 (17)	160 (13)
**Health status, n (%)**					
Chronic diseases (self-reported)					
none	6076 (52)	468 (78)	1386 (66)	3871 (50)	351 (29)
one	3979 (34)	116 (19)	604 (29)	2793 (36)	466 (39)
≥ 2	1545 (13)	24 (4)	121 (6)	1013 (13)	388 (32)
**Health behaviors, n(%)**					
Physical activity for at least 30 minutes					
≥ 5 days/week	6395 (55)	368 (61)	1330 (63)	4226 (55)	471 (39)
2-4 days/week	2481 (21)	109 (18)	396 (19)	1743 (23)	233 (19)
≤ 1 day/week	761 (7)	27 (5)	93 (4)	512 (7)	129 (11)
Smoking status					
never smoker	4453 (38)	238 (40)	802 (38)	2981 (39)	432 (36)
former smoker	5937 (51)	314 (52)	1121 (53)	3890 (51)	612 (51)
current smoker	789 (7)	37 (6)	128 (6)	530 (7)	94 (8)
Alcohol consumption					
abstainer	2122 (18)	78 (13)	258 (12)	1479 (19)	307 (25)
low risk	4911 (42)	248 (41)	920 (44)	3353 (44)	390 (32)
at risk	2977 (26)	188 (31)	685 (32)	1887 (25)	217 (18)

For peer review only - http://bmjopen.bmj.com/site/about/guidelines.xhtml

Table 1. Continued

**Characteristic**	**All**	**1. Excellent**	**2. Good**	**3. Moderate**	**4. Poor**
**n**	**11 600**	**602**	**2111**	**7677**	**1205**
**Biomarkers, n (%)**					
BMI[a] in kg / m^2					
<23	1323 (11)	107 (18)	295 (14)	822 (11)	99 (8)
≥ 23 & < 30[$]	8002 (69)	436 (72)	1560 (74)	5317 (69)	689 (57)
≥ 30	2264 (20)	64 (11)	256 (12)	1533 (20)	411 (34)
Blood pressure in mm Hg					
SBP ≤ 140/ 160[b] & DBP < 90[$]	6888 (59)	367 (61)	1271 (60)	4511 (59)	739 (61)
SBP ≤ 140/ 160[b] & DBP ≥ 90	92 (<1)	3 (<1)	20 (1)	64 (1)	5 (<1)
SBP > 140/ 160[b] & DBP < 90	3822 (33)	194 (32)	670 (32)	2560 (33)	398 (33)
SBP > 140/ 160[b] & DBP ≥ 90	774 (7)	42 (7)	145 (7)	528 (7)	59 (5)
CHOL/ HDL ratio					
< 3.5	5561 (48)	310 (51)	1040 (49)	3663 (48)	548 (45)
3.5-4.9[$]	4540 (39)	220 (37)	820 (39)	3022 (39)	478 (40)
> 5	1345 (12)	68 (11)	227 (11)	895 (12)	155 (13)
FEV1/ FVC ratio					
≥ 70[$]	8860 (76)	473 (79)	1625 (77)	5862 (76)	900 (75)
< 70	2740 (24)	134 (22)	486 (23)	1815 (24)	305 (25)
HbA1C in mmol/ mol (% of total Hb)					
< 48 (< 6.5%)[$]	9208 (79)	523 (87)	1767 (84)	6072 (79)	846 (70)
48-52 (6.5 -7%)	424 (4)	7 (1)	43 (2)	288 (4)	86 (7)
53-64 (7-8%)	324 (3)	0 (0)	39 (2)	217 (3)	68 (6)
> 64 (> 8%)	88 (1)	2 (<1)	7 (<1)	57 (1)	22 (2)
Hb in g/ dl (mmol/ L)					
< 12.1 / 13.7 (< 7.5/ 8.5) [c$]	886 (8)	46 (8)	166 (8)	549 (7)	125 (10)
≥ 12.1 / 13.7 (≥ 7.5/ 8.5) [c]	10 545 (91)	552 (92)	1921 (91)	7018 (91)	1054 (87)

For peer review only - http://bmjopen.bmj.com/site/about/guidelines.xhtml

Table 1. Continued

**Characteristic**	**All**	**1. Excellent**	**2. Good**	**3. Moderate**	**4. Poor**
**n**	**11 600**	**602**	**2111**	**7677**	**1205**
**Biomarkers, n (%)**					
TSH in mIU/L & fT4 in pmol/L					
TSH: 0.5-4.0 & fT4: 11-19.5[$]	2204 (19)	99 (16)	413 (20)	1466 (19)	226 (19)
TSH > 4.0 & fT4 ≥ 11 or <11	427 (4)	24 (4)	61 (3)	292 (4)	50 (4)
TSH < 0.5 & fT4 ≥ 11	81 (1)	6 (1)	8 (<1)	59 (1)	8 (1)
eGFR [d] in ml/min/1.73m^2					
≥ 90[$]	3809 (33)	179 (30)	622 (29)	2568 (33)	440 (37)
60-89	6577 (57)	375 (62)	1285 (61)	4315 (56)	602 (50)
45-59	898 (8)	40 (7)	166 (8)	594 (8)	98 (8)
< 45	151 (1)	4 (1)	14 (1)	98 (1)	35 (3)
HSI					
≤ 36[$]	2255 (19)	128 (21)	471 (22)	1486 (19)	170 (14)
> 36	1502 (4)	46 (8)	188 (9)	1031 (13)	237 (20)
MMSE score[e]					
25-30[$]	10738 (93)	552 (92)	1980 (94)	7178 (94)	1028 (85)
< 25	786 (7)	53 (9)	122 (6)	449 (6)	162 (14)
Sum score biomarkers					
none affected	600 (5)	33 (5)	132 (6)	386 (5)	49 (4)
≤ 2	6606 (57)	369 (61)	1298 (61)	4385 (57)	554 (46)
≥ 3	4394 (38)	202 (33)	670 (32)	2874 (37)	589 (49)

Notes: Blood based biomarkers are reported in the International System of Units (SI) followed by conventional units if used in database. Values marked with $ are cut offs used to define normal values. All percentages have been rounded off to the nearest whole number; percentages may not add up to 100% due to rounding and missing values. Missing percentages were 31% for FEV1/FVC ratio; 75% for TSH and fT4; 68% for HSI.

[a.] Cut-off was adjusted for age.

[b] Higher cutoff for SBP was used if participants were aged ≥80.

[c.] Cut offs are adjusted for sex, men had higher cut-off.

[d.] Calculated by the Cockcroft Gauld formula using serum creatinin in umol/l, age, weight, and adjusted for sex.

[e] Cut-offs are adjusted for level of education.

Abbreviations: BMI, body mass index; SBP, systolic blood pressure; DBP, diastolic blood pressure; CHOL, cholesterol; HDL, high-density lipoprotein; FEV1, forced expiration volume in 1 second; FVC, forced vital capacity; HbA1C, Hemoglobin A1C; Hb, hemoglobin; TSH, thyroid stimulating hormone; fT4, free thyroxine; eGFR, estimated glomeration filtration ratio; HSI, Hepatic Steatosis Index; MMSE, Mini Mental State Examination.

Table 2: Differences between completers and non-completers for baseline variables used in final model.

Characteristic	Completers N = 8590	Non-completers N = 3010	p-value
**Demographics**			
Age in years, median (IQR 25 - 75)[1]	68 (66 - 72)	69 (67 - 73)	<0.001
Male sex, n (%)[2]	4132 (48.1)	1352 (44.9)	0.001
Education, n (%)[2]			
low	4955 (57.7)	1608 (53.4)	<0.001
intermediate	1678 (19.5)	359 (11.9)	<0.001
high	1957 (22.8)	282 (9.4)	<0.001
missing percentage	0.5%	26%	
**Health status**			
Self-reported chronic diseases, n (%)[2]			
none	4435 (51.6)	1641 (54.5)	0.003
one	3023 (35.2)	956 (31.8)	0.004
≥ 2	1132 (13.2)	413 (13.7)	0.399
missing percentage	0%	0%	
**Health behaviors**			
Physical activity for at least 30 minutes, n (%)[2]			
≥ 5 days/ week	5732 (66.7)	663 (22.0)	<0.001
2-4 days/ week	2191 (25.5)	290 (9.6)	<0.001
≤ 1 day/ week	667 (7.8)	94 (3.1)	<0.001
missing percentage	0%	65%	
Smoking status, n (%)[2]			
never smoker	3349 (39.0)	1104 (36.7)	0.007
former smoker	4628 (53.9)	1309 (43.5)	<0.001
current smoker	613 (7.1)	176 (5.8)	0.007
missing percentage	0%	13%	
Alcohol consumption, n (%)[2]			
abstainer	1760 (20.5)	362 (12.0)	<0.001
low-risk alcohol consumption	4224 (49.2)	687(22.8)	<0.001
at risk alcohol consumption	2606 (30.3)	371 (12.3)	<0.001
missing percentage	0%	53%	
**Biomarkers[2]**			
≤ 2 affected	5859 (68.2)	1185 (39.4)	<0.001
≥ 3 affected	2731 (31.8)	1604 (53.3)	<0.001
missing percentage	0%	7%	

[1.] Equality of distributions was tested using the Wilcoxon Ranked Sum Test.

[2.] Equality of proportions was tested using the two sample test of proportions.

Abbreviations: n, number of participants; IQR, inter quartile range.

For peer review only

Trajectories of SRH over 5 years

Of all evaluated models, four trajectories of SRH over a five years period showed the best fit based on lowest Bayesian Information Criteria (BIC), Bayes factors, and adequate posterior diagnostics (Appendix C Tables C1 and C2). According to this basic model, 607 (5.6%), 2111 (18.8%), 7677 (65.3%), and 1205 (9.6%) people were assigned to the excellent, good, moderate, and poor SRH trajectory groups, respectively (Figure 1; Appendix C Figure C1).

Table 1 presents baseline characteristics of participants in all trajectory groups. People in poor SRH trajectories were on average older, more often female, lower educated, more often physically inactive, more often alcohol abstainer, and they had more self-reported chronic diseases compared to people belonging in the excellent, good and moderate SRH trajectory groups. Concerning objectively measured biomarkers, people in the poor SRH trajectory had higher BMI, less often high blood pressure, but more often high CHOL/HDL ratio, Hb levels, HSI index, and they scored lower on cognitive function compared to people in moderate, good and excellent SRH trajectory groups. In addition, people in poor SRH trajectory groups had more abnormal values of biomarkers compared to people in moderate, good and excellent SRH trajectory groups.

(Figure 1 here)

Identification of covariates of trajectory membership probability

Table 3 presents the results from multivariate logistic regression analyses on group membership of SRH. Model 2 performed worse compared to both models 1 and 3 (BIC: -61 952; AIC:1.810). The simplest model with only self-reported covariates (model 1) had lowest BIC (-62 498), but higher AIC (1.806) compared to model 3 that included a sum score of biomarkers as well (BIC:-61 729; AIC: 1.803).

For peer review only - http://bmjopen.bmj.com/site/about/guidelines.xhtml

Table 3. Regression estimates (relative risk ratios and 95% confidence intervals) of poor SRH relative to excellent SRH from multivariate logistic regression models on SRH trajectory group membership.

Covariate	Excellent SRH	Poor SRH trajectory Model 1[a]	Model 2[a]	Model 3[a]
Age	Ref.	1.01 (0.98; 1.04)	1.02 (0.99; 1.05)	1.01 (0.98; 1.04)
Sex,				
male	Ref.	Ref.	Ref.	Ref.
female	Ref.	1.42 (1.09; 1.86)	1.64 (1.24; 2.17)	1.45 (1.11; 1.89)
Education				
low	Ref.	Ref.	Ref.	Ref.
intermediate	Ref.	0.76 (0.54; 1.05)	0.77 (0.56 ; 1.07)	0.79 (0.57; 1.10)
high	Ref.	0.52 (0.37; 0.70)	0.58 (0.42; 0.79)	0.55 (0.40; 0.76)
Chronic diseases				
none	Ref.	Ref.	Ref.	Ref.
one	Ref.	7.80 (5.74; 10.60)	7.03 (5.16; 9.59)	7.76 (5.70; 10.57)
≥ 2	Ref.	26.35 (16.08; 43.20)	21.13 (12.81; 34.86)	25.03 (15.24; 41.09)
Physical activity for at least 30 minutes				
≥ 5 days/ week	Ref.	Ref.	Ref.	Ref.
2-4 days/ week	Ref.	1.64 (1.22; 2.19)	1.56 (1.16 ; 2.08)	1.61 (1.20; 2.15)
≤ 1 day/ week	Ref.	2.83 (1.75; 4.55)	2.55 (1.58 ; 4.13)	2.85 (1.76; 4.60)
Smoking status				
never	Ref.	Ref.	Ref.	Ref.
former	Ref.	1.43 (1.09; 1.88)	1.41 (1.08 ; 1.85)	1.42 (1.08; 1.86)
current	Ref.	1.78 (1.06; 2.96)	1.76 (1.05 ; 2.96)	1.71 (1.02; 2.89)
Alcohol consumption				
abstainer	Ref.	Ref.	Ref.	Ref.
low risk	Ref.	0.53 (0.38; 0.74)	0.56 (0.40; 0.79)	0.52 (0.37; 0.73)
at risk	Ref.	0.42 (0.29; 0.62)	0.44 (0.31; 0.66)	0.42 (0.29; 0.62)
Abnormal values of biomarkers[b]				
body composition	Ref.		1.34 (1.03; 1.76)	
cardiovascular function	Ref.		1.36 (1.06; 1.74)	
lung function	Ref.		1.12 (0.84; 1.50)	
glucose metabolism	Ref.		3.71 (1.68; 8.18)	
hematological cond.	Ref.		1.48 (0.95; 2.31)	
endocrine function	Ref.		0.96 (0.52; 1.77)	
renal function	Ref.		0.73 (0.56; 0.97)	
liver function	Ref.		1.80 (1.17; 2.77)	
cognitive function	Ref.		1.53 (1.00; 2.35)	
Sum score biomarkers				
≤ 2 affected	Ref.			Ref.
≥ 3 affected	Ref.			1.51 (1.16; 1.96)

[a]: Fit statistics: Model 1: n: 8679, AIC: 1.806, BIC: -62498; Model 2: n: 8679, AIC: 1.810, BIC:-61295; Model 3: n: 8590, AIC:1.803, BIC:-61729. Participants with missing data for covariates were excluded from the analysis.

[b] Participants with normal values of the biomarkers were used as the reference category.

Abbreviations: ref, reference category; n, number of participants; AIC, Akaike Information Criterium; BIC Bayesian Information Criterion.

For peer review only - http://bmjopen.bmj.com/site/about/guidelines.xhtml

However, both models had different sample sizes due to missing values for biomarkers in model 3. Taking into account the exploratory nature of this step in the analysis, type II error (an underfit model) would be more undesirable than type I error (an overfit model). Therefore the covariates included in model 3 were used for the final model (see Table 3, model 3).

Final model adjusted for associated covariates

The final trajectory model was modeled by jointly estimating the basic model and the covariates age, sex, educational level, self-reported chronic diseases, physical activity behavior, smoking behavior, alcohol consumption, and the sum score of affected biomarkers as risk factors. The final model assigned 471 (6.0%), 1727 (20.3%), 5628 (64.4%), and 764 (9.6%) people to the excellent, good, moderate, and poor SRH trajectories. The final model including covariates showed best fit statistics of posterior probability of group assignment (Table D3). The basic model overrepresented the proportion of older people in the poor and moderate groups, and underrepresented the proportion of people in the excellent and good trajectories, compared to the final model that took into account the effect of covariates (Table D3).

Table 4 presents the odds ratios of each of the risk factors independent of the level of other risk factors of people assigned to poor, moderate, and good SRH trajectory groups using the excellent SRH trajectory as reference category. Increasing number of chronic diseases increased the odds of membership in the poor SRH trajectory relative to the excellent SRH trajectory (OR: 10.4; 95% CI: 7.45 - 14.71 for one chronic disease, OR: 37.7; 95% CI 22.48 - 72.28 for two or more chronic diseases). Female gender, low education level, physical inactivity, (former) smoking, alcohol abstinence, and presence of 3 or more abnormal values of biomarkers increased the odds of the poor SRH trajectory membership relative to membership in the excellent SRH trajectory (Table 4).

For peer review only - http://bmjopen.bmj.com/site/about/guidelines.xhtml

Table 4. Odds ratios and 95% confidence intervals per predictor for being member of the good, moderate or poor SRH trajectory group relative to the excellent group (n = 8590[a]).

	Odds ratios (95% Confidence Interval)			
**Predictor**	**Exc. SRH**	**Good SRH**	**Moderate SRH**	**Poor SRH**
Age				
65-69	Ref.	Ref.	Ref.	Ref.
70-74	Ref.	0.99 (0.77; 1.33)	0.92 (0.75; 1.19)	1.02 (0.78; 1.41)
75-79	Ref.	1.38 (0.87; 2.27)	1.31 (0.88; 2.14)	1.32 (0.77; 2.32)
≥ 80	Ref.	1.14 (0.57; 2.48)	1.05 (0.59; 2.15)	1.09 (0.50; 2.55)
Sex,				
male	Ref.	Ref.	Ref.	Ref.
female[$]	Ref.	1.03 (0.78; 1.33)	1.21 (0.97; 1.58)	1.45 (1.09; 2.02)
Education				
low	Ref.	Ref.	Ref.	Ref.
intermediate[$]	Ref.	1.10 (0.79; 1.53)	0.87 (0.64; 1.17)	0.76 (0.52; 1.11)
high[$]	Ref.	0.96 (0.73; 1.32)	0.55 (0.42; 0.72)	0.48 (0.34; 0.69)
Chronic diseases				
none	Ref.	Ref.	Ref.	Ref.
one	Ref.	2.10 (1.55; 2.87)	3.55 (2.67; 4.78)	10.4 (7.45: 14.71)
≥ 2	Ref.	1.60 (0.91; 3.25)	5.28 (3.39; 9.73)	37.7 (22.48: 72.28)
Physical activity for at least 30 minutes				
≥ 5 days/ week	Ref.	Ref.	Ref.	Ref.
2-4 days/ week[$]	Ref.	0.99 (0.74; 1.32)	1.35 (1.06; 1.78)	1.61 (1.15; 2.16)
≤ 1 day/ week	Ref.	0.95 (0.57; 1.71)	1.42 (0.95; 2.44)	3.12 (1.81; 5.33)
Smoking status				
never	Ref.	Ref.	Ref.	Ref.
former[#]	Ref.	1.08 (0.83; 1.40)	1.18 (0.95; 1.46)	1.52 (1.18; 2.06)
current[$]	Ref.	1.09 (0.66; 1.99)	1.47 (0.92; 2.55)	1.87 (1.04; 3.44)
Alcohol consumption				
abstainer	Ref.	Ref.	Ref.	Ref.
low risk	Ref.	1.39 (0.95; 2.00)	0.89 (0.63; 1.19)	0.54 (0.36; 0.77)
at risk	Ref.	1.39 (0.90; 2.09)	0.70 (0.50; 0.98)	0.41 (0.26; 0.65)
Sum score biomarkers				
≤ 2 affected	Ref.	Ref.	Ref.	Ref.
≥ 3 affected	Ref.	0.89 (0.66; 1.17)	1.92 (0.87; 1.42)	1.50 (1.10; 1.97)

Final trajectory model including identified predictors of SRH trajectory membership by multinomial logistic regression analysis (table 2, model 3) adjusted for age (5 year intervals from 65 years old), education, and sex.
[a] 3010 of 11.600 participants aged 65 years and older were excluded from the analysis due to missing data on covariates included in the final model.
$ Wald tests showed no differences between poor and moderate SRH trajectories
# Wald tests showed no differences between moderate and good SRH trajectories
Abbreviations: Exc., excellent; Ref., reference category; SRH, self-rated health.

For peer review only - http://bmjopen.bmj.com/site/about/guidelines.xhtml

Wald tests implied that all trajectory groups were distinguished by the number of self-reported chronic diseases, alcohol consumption, and the sum score of affected biomarkers (p-values <0.001).

Sensitivity analysis including alteration of the composite measure for multimorbidity without anxiety and depressive disorders did not alter trajectory group sizes, shapes, and odds ratios (results not shown). Dual trajectory modeling accounting for non-random attrition showed constant annual attrition probabilities between 10% (good SRH) and 17% (poor SRH) for all trajectory groups (Appendix D, Figure D1). Posterior probability of group assignment did not improve when modeling the trajectories accounting for attrition bias (Appendix D, table D1).

DISCUSSION

In this sample of an ongoing large cohort study of Dutch community-dwelling older adults, four stable trajectories of SRH over five years were identified. The majority (65.3%) of the participants were classified into the moderate SRH category, followed by good (18.8%), poor (10.2%), and excellent (5.6%) SRH. The results of our study confirmed our a priori hypothesis that poor SRH was associated with multimorbidity, health risk behaviors, and abnormalities in biomarkers. The number of chronic diseases seems to be one of the key factors that determines someone's SRH trajectory membership, as this was the only covariate under consideration that was significantly associated with membership of all SRH trajectories. In addition, poor SRH trajectory membership was associated with being female, a low education level, health risk behaviors, and presence of three or more affected biomarkers.

Contrary to previous studies investigating trajectories of SRH, this study identified only stable trajectories of self-rated health of older community-dwelling adults during five years (5–7,39). Other studies with comparable measurement intervals, and study duration identified the

For peer review only - http://bmjopen.bmj.com/site/about/guidelines.xhtml

majority of their participants in the stable trajectories as well, however they also identified small groups with declining and improving trajectories (5,7). Sample size was not the limiting factor to identify more groups, however, the posterior diagnostic criteria became worse when adding more than four trajectory groups, indicating four groups was the optimum for our sample. Participants of the current study were older than the populations used in other studies investigating trajectories of SRH. Response shift in SRH is known to occur among older adults (40). Compared to their younger counterparts, older adults are suggested to base their SRH more on psychological and life-style behaviors, and less on functional status and physical health, which might indicate reprioritization response shift (41,42). Furthermore, older adults adapt their standards of good health over time, also known as recalibration response shift (40), which can explain the stable trajectories of SRH over time in the present study sample.

Consistent with other studies investigating trajectories of SRH, we found strong associations between increasing numbers of baseline self-reported chronic diseases and poor SRH trajectories (5–7). When participants reported only one chronic disease, they had a two, three-and-half, and ten times higher odds of being a member of good, moderate, and poor SRH trajectory compared to the excellent SRH trajectory, respectively. People suffering two or more self-reported chronic diseases were 38 times more likely to be in the poor SRH trajectory group rather than the excellent SRH trajectory group. Earlier studies found weaker associations between poorer SRH trajectories and number of chronic diseases (6,7). The difference in results might be explained by the different number and combinations of covariates used as predictors in different studies. For instance, previous studies focused on chronic physical health disorders to calculate a composite measure of multimorbidity (5,6). For this study, the eleven most burdensome chronic diseases forecasted for the next decades

For peer review only

For peer review only - http://bmjopen.bmj.com/site/about/guidelines.xhtml

by the Dutch National Institute for Public Health and the Environment were used to measure chronic diseases, which included depression and anxiety disorders. The inclusion of depression and anxiety disorders in our composite measure of chronic diseases may have led to the strong associations between self-rated chronic diseases and poor SRH trajectories in the present study, because depressive symptoms are considered a risk factor for poor SRH (43). However, sensitivity analyses excluding depression and anxiety disorders in the composite score for chronic diseases led to similar results. Therefore, it is not expected that the differences in composite measures for chronic diseases explain the differences in magnitude of odds for membership in the poor SRH trajectory with increasing number of chronic diseases found in the present study compared to previous studies.

Strengths of this study are the large sample size, and short measurement intervals for SRH that contribute to the robustness of the findings. In addition, the use of biomarkers next to self-reported data was, to the best of our knowledge, not previously investigated in combination with trajectory analyses. There were limitations as well. Firstly, although we found a strong association between self-reported diseases and poorer SRH trajectories, we cannot rule out reverse causation. The presented odds ratios only measure relative change on group level and are not suited to generalize to individual probability of group membership. It is therefore hard to translate these results into concrete clinical implications, as there will always be people having multimorbidity combined with excellent self-rated health. Second, in this older population, the use of self-reported measurements used for measuring the number of chronic diseases may have led to an over- or underestimation of the prevalence of diseases due to non-differential misclassification bias. Finally, attrition may have threatened the generalizability of our results (44). However, sensitivity analysis with trajectories jointly modeled with attrition (45) did not improve group allocation probabilities. In addition,

For peer review only - http://bmjopen.bmj.com/site/about/guidelines.xhtml

constant annual attrition probabilities below 20% for all groups were identified, which led us to conclude that attrition rates were constant among all trajectory groups.

IMPLICATIONS AND CONCLUSIONS

The present study identified four stable trajectories of SRH over five years in Dutch community-dwelling, older adults where the majority of the sample had moderate SRH. Being female, lower levels of education, health risk behaviors (smoking, physical inactivity, and alcohol abstinence), and presence of three or more abnormal biomarkers were associated with poor SRH trajectory membership. The identified modifiable determinants may provide a basis for future preventive strategies.

For peer review only

For peer review only - http://bmjopen.bmj.com/site/about/guidelines.xhtml

DECLARATIONS

Ethical considerations

The Lifelines Cohort study was approved by the research ethics committee of the University Medical Center Groningen, The Netherlands (registration number: 2007/152). All participants provided written informed consent before study enrollment.

Availability of data

The data are available from www.lifelines.nl. Researchers interested in queries related to data access may contact the Lifelines Research Office via data@lifelines.nl.

Funding

This work was supported by the University of Groningen, in collaboration with the University Medical Center of Groningen, departments of epidemiology and internal medicine and geriatrics. The Lifelines Biobank initiative was funded by Fonds Economische Structuurversterking (FES), Samenwerkingsverband Noord Nederland (SNN), and Ruimtelijk Economisch Programma (REP). JMV is funded through the Netherlands Organization for Health Research and Development (NWO-ZonMw), grant number 91619060.

Conflict of interest

None

Acknowledgements

The authors wish to acknowledge the service of the Lifelines Cohort Study and all study participants.

Patient and public involvement statement

This research as well as the Lifelines Cohort Study database development was performed without public or patient involvement.

For peer review only - http://bmjopen.bmj.com/site/about/guidelines.xhtml

Author contributions

NS obtained funding and supervised the project. MF performed statistical analyses and wrote the first draft of the manuscript. NS and JMV aided in interpreting the results. All authors were involved in the study design, revising manuscript draft for important intellectual content, and gave approval for the final manuscript, and thereby taking full responsibility for the work and manuscript content.

For peer review only

For peer review only - http://bmjopen.bmj.com/site/about/guidelines.xhtml

REFERENCES

1. Jylhä M. What is self-rated health and why does it predict mortality? Towards a unified conceptual model. *Soc Sci Med* 2009;69(3):307–16.

2. DeSalvo KB, Bloser N, Reynolds K, et al. Mortality Prediction with a Single General Self-Rated Health Question A Meta-Analysis. *J Gen Intern Med* 2005;20:267–75.

3. Idler EL, Benyamini Y. Self-Rated Health and Mortality : A Review of Twenty-Seven Community Studies. *J Health Soc Behav* 1997;38(1):21–37.

4. Nagin DS, Odgers CL. Group-based trajectory modeling in clinical research. *Annu Rev Clin Psychol* 2010;6:109–38.

5. Schmitz N, Gariépy G, Smith KJ, et al. Trajectories of self-rated health in people with diabetes: Associations with functioning in a prospective community sample. *PLoS One* 2013;8(12):1–7.

6. Lee HL, Huang HC, Lee M Der, et al. Factors affecting trajectory patterns of self-rated health (SRH) in an older population-A community-based longitudinal study. *Arch Gerontol Geriatr* 2012;54(3):334–41.

7. Ayyagari P, Ullrich F, Malmstrom TK, et al. Self-Rated Health Trajectories in the African American Health Cohort. *PLoS One* 2012;7(12).

8. FDA-NIH Biomarker Working Group. BEST (Biomarkers, EndpointS, and other Tools) Resource. Silver Spring: Food and Drug Administration; Bethesda: National Institutes of Health. 2016.

9. Stenholm S, Pentti J, Kawachi I, et al. Self-rated health in the last 12 years of life

For peer review only - http://bmjopen.bmj.com/site/about/guidelines.xhtml

compared to matched surviving controls: The health and retirement study. *PLoS One* 2014;9(9): e107879.

10. Stenholm S, Kivimäki M, Jylhä M, et al. Trajectories of self-rated health in the last 15 years of life by cause of death. *Eur J Epidemiol* 2016;31(2):177–85.

11. Scholtens S, Smidt N, Swertz MA, et al. Cohort Profile: LifeLines, a three-generation cohort study and biobank. *Int J Epidemiol* 2015;44(4):1172–80.

12. Ware JE, Sherbourne CD. The MOS 36-item short-form health survey (SF-36). I. Conceptual framework and item selection. *Med Care* 1992 Jun;30(6):473–83.

13. Aaronson NK, Muller M, Cohen PD, et al. Translation, validation, and norming of the Dutch language version of the SF-36 Health Survey in community and chronic disease populations. *J Clin Epidemiol* 1998 Nov;51(11):1055–68.

14. Unesco Institute for Statistics. International Standard Classification of Education ISCED 2011. Montreal: UNESCO Institute for Statistics; 2012. 88p.

15. Centraal Bureau voor de Statistiek. SOI 2016, Standaard Onderwijsindeling 2016. Den Haag / Heerlen: CBS; 2017. 40p.

16. Kemper HCG, Ooijendijk WTM, Stiggelbout M. Consensus over de Nederlandse norm voor gezond bewegen. *Tijdschr Soc Gezondheidsz* 2000;78(3):180–3.

17. Gezondheidsraad. Richtlijnen goede voeding 2015. Den Haag: Gezondheidsraad; 2015. 94 p. Reportno.: 2015/24.

18. Mathers J, Deary I, Kuh D, et al. Guidelines for biomarkers of healthy ageing. 93p. https://mrc.ukri.org/documents/pdf/biomarkers-of-healthy-ageing/ (accessed 4 October

For peer review only - http://bmjopen.bmj.com/site/about/guidelines.xhtml

2018)

19. Winter JE, Macinnis RJ, Wattanapenpaiboon N, et al. BMI and all-cause mortality in older adults: a meta-analysis. *Am J Clin Nutr* 2014;99:875–90.

20. Mannino DM, Diaz-Guzman E. Interpreting lung function data using 80% predicted and fixed thresholds identifies patients at increased risk of mortality. *Chest* 2012;141(1):73–80.

21. Sorino C, Sherrill D, Guerra S, et al. Prognostic value of FEV1/FEV6 in elderly people. *Clin Physiol Funct Imaging* 2011;31(2):101–7.

22. Fried LP, Xue QL, Cappola AR, et al. Nonlinear multisystem physiological dysregulation associated with frailty in older women: Implications for etiology and treatment. *J Gerontol A Biol Sci Med Sci* 2009;64(10):1049–57.

23. Rodondi N, Den Elzen WPJ, Bauer DC, et al. Subclinical Hypothyroidism and the Risk of Coronary Heart Disease and Mortality. *JAMA* 2010;304(12):1365–74.

24. Pearce SHS, Razvi S, Yadegarfar ME, et al. Serum Thyroid Function, Mortality and Disability in Advanced Old Age: The Newcastle 85+ Study. *J Clin Endocrinol Metab* 2016;101(11):4385–94.

25. Mammen JS, McGready J, Ladenson PW, et al. Unstable Thyroid Function in Older Adults Is Caused by Alterations in Both Thyroid and Pituitary Physiology and Is Associated with Increased Mortality. *Thyroid* 2017;27(11):1370–77.

26. Montesanto A, De Rango F, Berardelli M, et al. Glomerular filtration rate in the elderly and in the oldest old: Correlation with frailty and mortality. *Age* 2014;36(3):1503–14.

For peer review only

For peer review only - http://bmjopen.bmj.com/site/about/guidelines.xhtml

27. Levey AS, Stevens LA, Schmid CH, et al. A new equation to estimate glomerular filtration rate. *Ann Intern Med* 2009;150(9):604–12.

28. Levey AS, Stevens LA. Estimating GFR using the CKD Epidemiology Collaboration (CKD-EPI) creatinine equation: more accurate GFR estimates, lower CKD prevalence estimates, and better risk predictions. *Am J Kidney Dis* 2010;55(4):622–7.

29. Kahl S, Strassburger K, Nowotny B, et al. Comparison of Liver Fat Indices for the Diagnosis of Hepatic Steatosis and Insulin Resistance. *PLoS One* 2014;9(4):e94059.

30. Lee J, Kim D, Jung H, et al. Hepatic steatosis index : A simple screening tool reflecting nonalcoholic fatty liver disease. *Dig Liver Dis* 2010;42(7):504–9.

31. Schmand B, Lindeboom J, Hooijer C, et al. Relation between education and dementia : the role of test bias revisited. *J Neurol Neurosurg psychiatry* 1995;59:170–4.

32. Nagin DS, Jones BL, Lima Passos V, et al. Group-based multi-trajectory modeling. *Stat Methods Med Res* 2016; 0(0):1-9.

33. Schwarz G. Estimating the dimension of a model. *Ann Stat* 1978;6(2):461–4.

34. Wasserman L. Bayesian Model Selection and Model Averaging. *J Math Psychol* 2000;44:92–107.

35. Nagin D. Group-based modeling of development. Cambridge, Massachusetts: Harvard University Press; 2005. 201 p.

36. Dziak JJ, Coffman DL, Lanza ST, et al. Sensitivity and specificity of information criteria. *Briefings in Bioinformatics* 2019; 00(0):1–13.

37. Jones BL, Nagin DS. A Stata plugin for estimating group-based trajectory models.

For peer review only - http://bmjopen.bmj.com/site/about/guidelines.xhtml

Pittsburgh; 2012

38. Jones BL, Nagin DS. A Note on a Stata Plugin for Estimating Group-based Trajectory Models. *Sociol Methods Res* 2013;42(4):608–13.

39. Sacker A, Wiggins RD, Bartley M, et al. Self-rated health trajectories in the United States and the United Kingdom: A comparative study. *Am J Public Health* 2007;97(5):812–8.

40. Spuling SM, Wolff JK, Wurm S. Response shift in self-rated health after serious health events in old age. *Soc Sci Med* 2017;192:85–93.

41. Hoeymans N, Feskens EJM, Kromhout D, et al. Aging And The Relationship Between Functional Status And Self-Rated Health In Elderly Men. *Soc Sci Med* 1997;45(10):1527–36.

42. Leinonen R, Heikkinen E, Jylhä M. Self-rated health and self-assessed change in health in elderly men and women - A five-year longitudinal study. *Soc Sci Med* 1998;46(4–5):591–7.

43. Han B. Depressive Symptoms and Self-Rated Health in Community-Dwelling Older Adults: A Longitudinal Study. *J Am Geriatr Soc* 2002;50:1549–56.

44. Genbäck M, Ng N, Stanghellini E, et al. Predictors of decline in self‐reported health : addressing non‐ignorable dropout in longitudinal studies of aging. *Eur J Ageing* 2018;15(2):211–20.

45. Haviland AM, Jones BL, Nagin DS. Group-based Trajectory Modeling Extended to Account for Nonrandom Participant Attrition. *Sociol Methods Res* 2011;40(2):367–90.

For peer review only - http://bmjopen.bmj.com/site/about/guidelines.xhtml

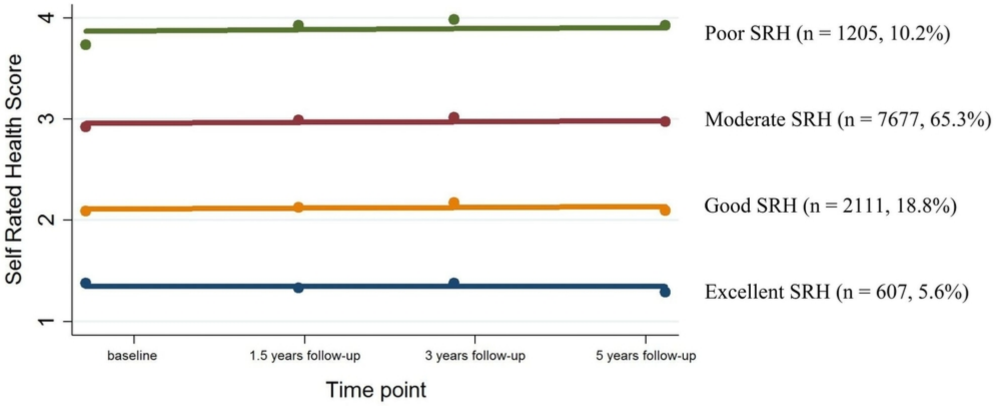

Figure 1: Non adjusted trajectories of SRH over five years using 11 600 adults aged 65 years and older of the Lifelines Cohort Study.

84x34mm (300 x 300 DPI)

APPENDIX A: BIOMARKER SPECIFICATION

Table A1: Cut-offs used to define normal and affected values for biomarkers per organsystem.

**Lung function**		
FEV1 (L) /FVC (L) ratio (multiplied by 100%)	Cut-off scores used (Quanjer et al., 2012):<br>Normal: ≥ 70%<br>Affected < 70%	
**Renal function**		
eGFR (in ml/min/1.73m2)	Estimated with the Cockcroft Gauld formula using serum creatine in umol/l (adjusted for age, sex, weight) (Cockcroft & Gault, 1976).<br>Cut-off scores used (Traynor, Mactier, Geddes, & Fox, 2006):<br>Normal: ≥90 ml/min/1.73m^2<br>Affected: <90 ml/min/1.73m^2	
**Endocrine function**[1]		
TSH (mIU/L)	Normscores (lab standards UMCG):<br>Low: <0.5 mIU/L<br>Normal: 0.5 – 4.0 mIU/L<br>High: ≥ 4.0 mIU/L	
fT4 (pmol/L)	Normscores (Boesten et al., 2012):<br>Low: < 11.0 pmol/L<br>Normal: 11.0 – 19.5 pmol/L<br>High: > 19.5 pmol/L	
**Immune function**		
Hb (mmol/L)	Different cut-offs used for men and women (lab standards UMCG).<br>Cut off used:<br>Men:<br>Normal: ≥8.5 and ≤ 11 mmol/L<br>Affected: <8.5, >11 mmol/L	Women:<br>Normal: ≥7.5 and ≤ 10 mmol/L<br>Affected: <7.5, >10 mmol/L
**Liver function**		
Hepatic Steatosis Index	Cut off used (Lee et al., 2010; Meems et al., 2015):<br>Normal: ≤ 36<br>Affected >36	
**Cognitive function**		
MMSE	Adjusted for level of education: primary education or less (max.6 years) and secondary or higher (>6 years) (Schmand, Lindeboom, Hooijer, & Jonker, 1995).<br>Cut-off used:<br>≤ Primary:<br>Normal: ≥25<br>Affected: <25	≥ Secondary:<br>Normal ≥27<br>Affected: <27
**Body composition**		
BMI (for Caucasian)	Age adjusted BMI cutoffs were used (Winter, Macinnis, Wattanapenpaiboon, & Nowson, 2014).<br>Cut offs used:<br>Normal: ≥23.0 BMI <30<br>Affected: <23 & BMI ≥30	
**Cardiovascular function**[2]		

For peer review only - http://bmjopen.bmj.com/site/about/guidelines.xhtml

SBP (mmHg)	Adjusted for age ((European Society of Hypertension/ European Society of Cardiology, 2014))<br>Cut-offs used:<br>Aged <80: Normal: ≤ 140 mmHg; High: >140 mmHg<br>Aged ≥80: Normal: ≤ 160 mmHg; High: >160 mmHg
DBP (mmHg)	Cut-off used (European Society of Hypertension/ European Society of Cardiology, 2014):<br>Normal: <90 mmHg<br>High: ≥90 mmHg
Total cholesterol (mmol/L)/ HDL (mmol/L) ratio	Cut offs used (European Society of Cardiology / European Atherosclerosis Society, 2016; Landelijke werkgroep Cardiovasculair risicomanagement, 2012):<br>Normal: <5.0<br>High ≥ 5.0
**Glucose metabolism**	
HbA1c (mmol (HbA1c) / mol (Hb))	Cut offs used (Fried et al., 2009):<br>Normal: < 48 mmol/mol (corresponding to 6,5% of total Hb)<br>Affected: ≥ 48 mmol/mol

Abbreviations: FER, forced expiratory ratio; eGFR, estimated glomerular filtration rate; TSH, thyroid stimulating hormone; FSH, follicle-stimulating hormone; HB, hemoglobin; MMSE, mini mental state examination; BMI, body mass index; SBP, systolic blood pressure; DBP, diastolic blood pressure; HDL, high density lipoprotein; HbA1C, glycated hemoglobin.

1. TSH cut-offs were interpreted with fT4; both TSH and fT4 should be in the normal range to score 'normal' concerning the endocrine system.

2. Blood pressure was interpreted with cholesterol levels; both diastolic and systolic bloodpressure and cholesterol/HDL ratio or should in the normal range to score 'normal' concerning the cardiovascular system.

References:

Boesten, L. S. M., Brugts, M. P., & van Rossum, A. P. (2012). Schildklierdiagnostiek : discrepantie FT4 en TSH. *Nederlands Tijdschrift Voor Geneeskunde*, *156*, A4167V.

Cockcroft, D. W., & Gault, H. (1976). Prediction of Creatinine Clearance from Serum Creatinine. *Nephron*, *16*(1), 31–41. European Society of Cardiology / European Atherosclerosis Society. (2016). 2016 ESC/EAS guidelines for the management of dyslipidemias. *European Heart Journal*, *37*, 2999–3058.

European Society of Hypertension/ European Society of Cardiology. (2014). 2014 guidelines for the management of arterial hypertension. *Primary Care Cardiovascular Journal*, *7*(2), 85–88.

Fried, L. P., Xue, Q. L., Cappola, A. R., Ferrucci, L., Chaves, P., Varadhan, R., … Bandeen-Roche, K. (2009). Nonlinear multisystem physiological dysregulation associated with frailty in older women: Implications for etiology and treatment. *Journals of Gerontology - Series A Biological Sciences and Medical Sciences*, *64*(10), 1049–1057.

Landelijke werkgroep Cardiovasculair risicomanagement. (2012). Dutch standard of cardiovasculair riskmanagement. *Huisarts Wet*, *55*(1), 14–28.

Lee, J., Kim, D., Jung, H., Lee, C., In, J., Kim, W., … Lee, H. (2010). Hepatic steatosis index : A simple screening tool reflecting nonalcoholic fatty liver disease. *Digestive and Liver Disease*,

For peer review only - http://bmjopen.bmj.com/site/about/guidelines.xhtml

42(7), 504–509.

Meems, L. M. G., De Borst, M. H., Postma, D. S., Vonk, J. M., Kremer, H. P. H., Schuttelaar, M. L. A., … De Boer, R. A. (2015). Low levels of Vitamin D are associated with multimorbidity: Results from the LifeLines Cohort Study. *Annals of Medicine*, *47*(6), 474–481.

Quanjer, P. H., Stanojevic, S., Cole, T. J., Baur, X., Hall, G. L., Culver, B. H., … Schindler, C. (2012). Multi-ethnic reference values for spirometry for the 3-95-yr age range: The global lung function 2012 equations. *European Respiratory Journal*, *40*(6), 1324–1343.

Schmand, B., Lindeboom, J., Hooijer, C., & Jonker, C. (1995). Relation between education and dementia : the role of test bias revisited. *Journal of Neurology, Neurosurgery and Psychiatry*, *59*, 170–174.

Traynor, J., Mactier, R., Geddes, C. C., & Fox, J. G. (2006). How to measure renal function in clinical practice: Estimated glomerular filtration rate in general practice. *Bmj*, *333*(7574), 918.2.

Winter, J. E., Macinnis, R. J., Wattanapenpaiboon, N., & Nowson, C. A. (2014). BMI and all-cause mortality in older adults: a meta-analysis. *Am J Clin Nutr*, *99*, 875–890.

For peer review only

For peer review only - http://bmjopen.bmj.com/site/about/guidelines.xhtml

APPENDIX B: FLOWCHART OF STUDY SAMPLE

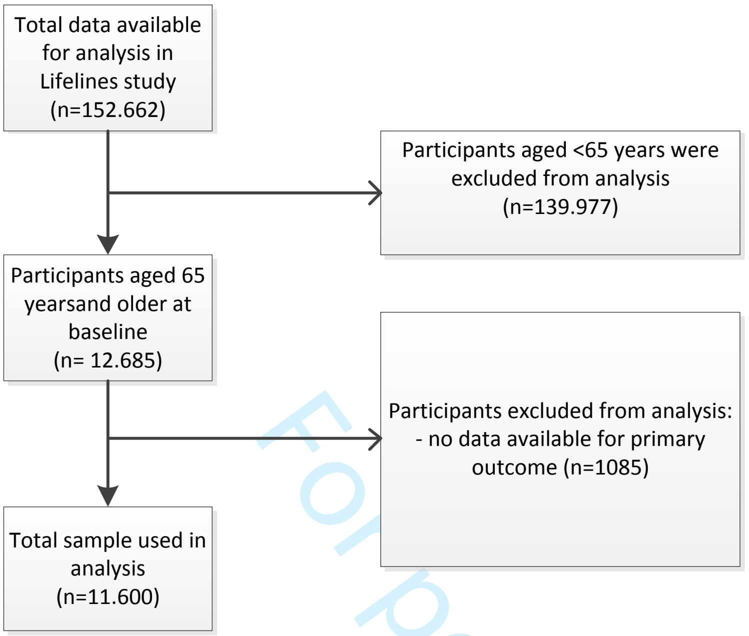

Figure B1. Flow of selection of study sample.

For peer review only

APPENDIX C: BASIC MODEL SPECIFICATIONS

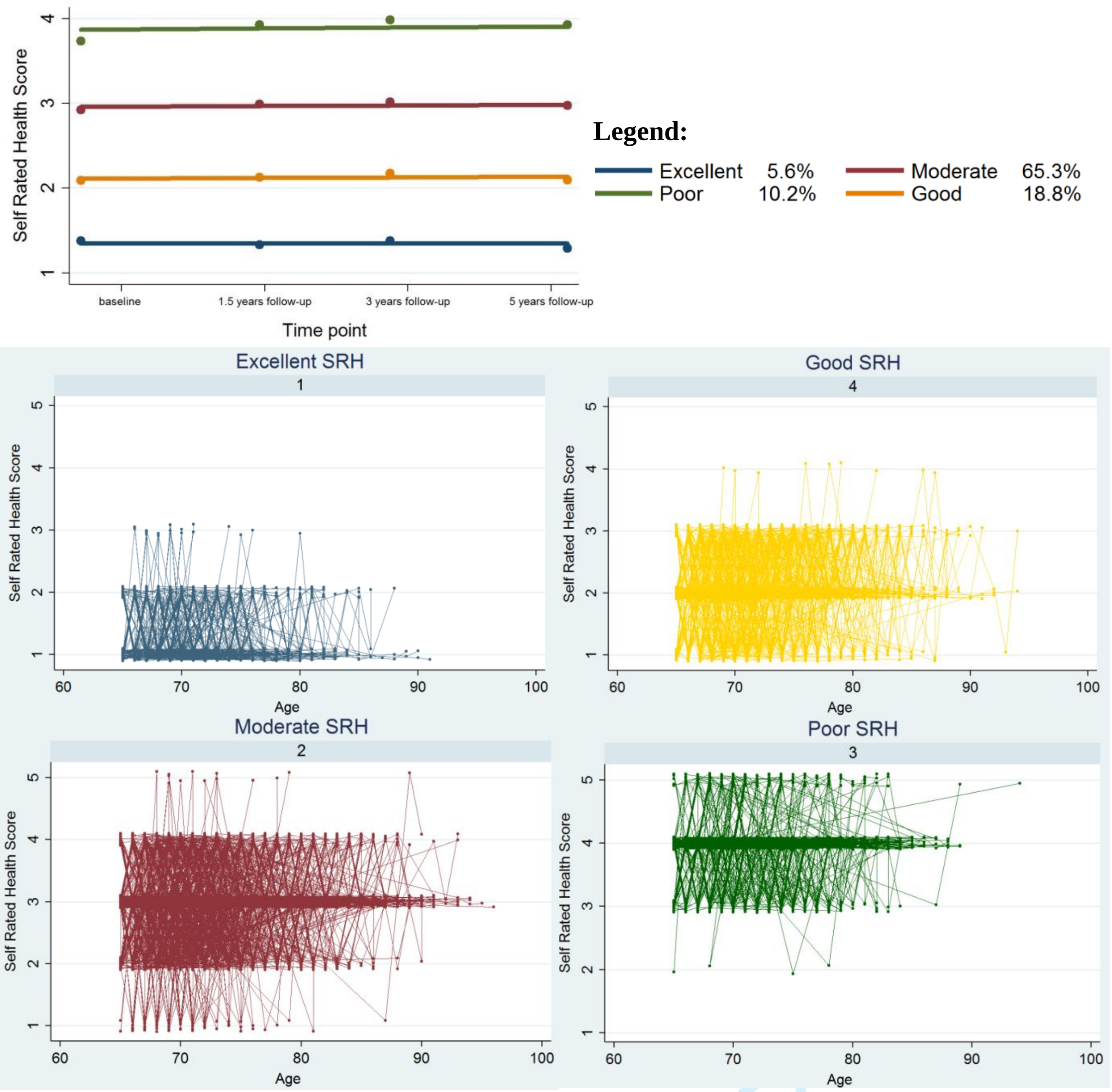

Figure C1: Basic mean trajectory groups of SRH (a), and observed individual trajectories per trajectory group (b-e) over five years of 11.600 people aged 65 years and older of the Lifelines Cohort. a. Dots represent the mean observed value per measurement moment; solid lines represent fit lines; dotted lines represent 95% confidence intervals of the fit lines.
b-e. Jittering was used for adding random noise to make all individual scores integer to avoid overlap of individual trajectories for people with identical trajectories.

For peer review only - http://bmjopen.bmj.com/site/about/guidelines.xhtml

Table C1: Bayesian Information Criteria and probability estimation Jeffreys's scale of evidence for Bayes factors of crude trajectory calculations with fixed quadratic growth terms used to select adequate number of groups.

**No. of groups**	**BIC** (n=11.600)	**Probability correct model**
2	-35016.79	0
3	-33001.46	0
4	-32240.77	0.98
5	-32259.49	< 0.01
6	-32278.20	< 0.01
7	-32244.54	0.02

Table C2: Posterior diagnostics of model performance of basic trajectory model.

**Group**	**Model estimate** (*π^*)	**95% CI**	**Proportion classified** (*p^*)	**Ave. PP**	**Odds correct classification**
1	.056	(.050; .062)	.052	.892	138.3
2	.653	(.642; .664)	.662	.941	8.4
3	.102	(.095; .109)	.104	.852	50.7
4	.188	(.179; .198)	.182	.863	27.1

For peer review only - http://bmjopen.bmj.com/site/about/guidelines.xhtml

APPENDIX D: SENSITIVITY ANALYSES

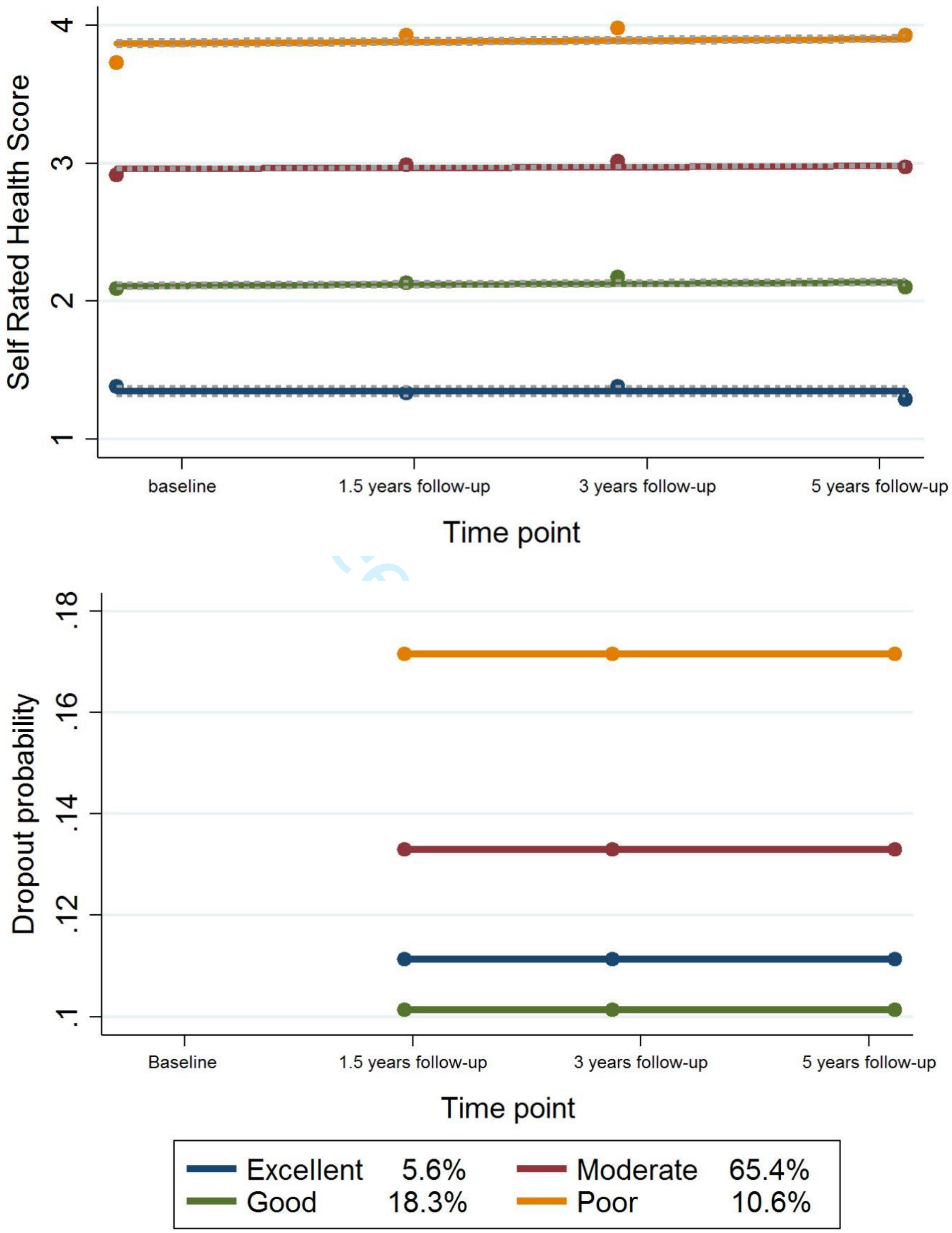

Figure D1: Trajectories of SRH jointly modelled with attrition.

For peer review only - http://bmjopen.bmj.com/site/about/guidelines.xhtml

Table D1. Comparison of posterior probability of assignment for the basic model, the model including covariates / risk factors, and the trajectory model that jointly modelled attrition (sensitivity analysis).

Group Allocation	N (%)	Excellent	Good	Moderate	Poor
**Basic model (step 1): posterior probability of assignment**					
Excellent	607 (5.6)	**0.89**	<0.01	0.01	0.05
Good	2111 (18.8)	0.11	**0.86**	0.06	<0.01
Moderate	8762 (65.3)	<0.01	0.15	**0.91**	0.08
Poor	1205 (10.2)	<0.01	<0.01	0.03	**0.85**
**Model with covariates (step 3): posterior probability of assignment**					
Excellent	471 (6.0)	**0.91**	0.05	<0.01	<0.01
Good	1727 (20.3)	0.09	**0.87**	0.04	<0.01
Moderate	5628 (64.4)	<0.01	0.08	**0.95**	0.09
Poor	764 (9.6)	<0.01	<0.01	0.02	**0.91**
**Model with attrition (sensitivity analysis): posterior probability of assignment**					
Excellent	609 (5.7)	**0.90**	0.05	0.01	<0.01
Good	2123 (18.7)	0.10	**0.86**	0.05	<0.01
Moderate	8762 (65.3)	<0.01	0.09	**0.91**	0.14
Poor	1191 (10.3)	<0.01	<0.01	0.04	**0.86**

*Rows may add to more than 1.0 due to rounding.

For peer review only

Reporting checklist for cohort study.

Based on the STROBE cohort guidelines.

Instructions to authors

Complete this checklist by entering the page numbers from your manuscript where readers will find each of the items listed below.

Your article may not currently address all the items on the checklist. Please modify your text to include the missing information. If you are certain that an item does not apply, please write "n/a" and provide a short explanation.

Upload your completed checklist as an extra file when you submit to a journal.

In your methods section, say that you used the STROBE cohortreporting guidelines, and cite them as:

von Elm E, Altman DG, Egger M, Pocock SJ, Gotzsche PC, Vandenbroucke JP. The Strengthening the Reporting of Observational Studies in Epidemiology (STROBE) Statement: guidelines for reporting observational studies.

		Reporting Item	Page Number
**Title and abstract**			
Title	#1a	Indicate the study's design with a commonly used term in the title or the abstract	1
Abstract	#1b	Provide in the abstract an informative and balanced summary of what was done and what was found	2
**Introduction**			
Background / rationale	#2	Explain the scientific background and rationale for the investigation being reported	4
Objectives	#3	State specific objectives, including any prespecified hypotheses	4
**Methods**			
Study design	#4	Present key elements of study design early in the paper	5
Setting	#5	Describe the setting, locations, and relevant dates, including	5

For peer review only

		periods of recruitment, exposure, follow-up, and data collection	
Eligibility criteria	#6a	Give the eligibility criteria, and the sources and methods of selection of participants. Describe methods of follow-up.	5
Eligibility criteria	#6b	For matched studies, give matching criteria and number of exposed and unexposed	n/a, not matched
Variables	#7	Clearly define all outcomes, exposures, predictors, potential confounders, and effect modifiers. Give diagnostic criteria, if applicable	5-6
Data sources / measurement	#8	For each variable of interest give sources of data and details of methods of assessment (measurement). Describe comparability of assessment methods if there is more than one group. Give information separately for for exposed and unexposed groups if applicable.	Appendix A1
Bias	#9	Describe any efforts to address potential sources of bias	8
Study size	#10	Explain how the study size was arrived at	9
Quantitative variables	#11	Explain how quantitative variables were handled in the analyses. If applicable, describe which groupings were chosen, and why	6-7
Statistical methods	#12a	Describe all statistical methods, including those used to control for confounding	6-8
Statistical methods	#12b	Describe any methods used to examine subgroups and interactions	6-8
Statistical methods	#12c	Explain how missing data were addressed	8
Statistical methods	#12d	If applicable, explain how loss to follow-up was addressed	8
Statistical methods	#12e	Describe any sensitivity analyses	8
**Results**			
Participants	#13a	Report numbers of individuals at each stage of study—eg numbers potentially eligible, examined for eligibility,	8

For peer review only

		confirmed eligible, included in the study, completing follow-up, and analysed. Give information separately for for exposed and unexposed groups if applicable.	
Participants	#13b	Give reasons for non-participation at each stage	Appendix B
Participants	#13c	Consider use of a flow diagram	Appendix B
Descriptive data	#14a	Give characteristics of study participants (eg demographic, clinical, social) and information on exposures and potential confounders. Give information separately for exposed and unexposed groups if applicable.	8, 12
Descriptive data	#14b	Indicate number of participants with missing data for each variable of interest	9-11
Descriptive data	#14c	Summarise follow-up time (eg, average and total amount)	8
Outcome data	#15	Report numbers of outcome events or summary measures over time. Give information separately for exposed and unexposed groups if applicable.	9-11
Main results	#16a	Give unadjusted estimates and, if applicable, confounder-adjusted estimates and their precision (eg, 95% confidence interval). Make clear which confounders were adjusted for and why they were included	14-16
Main results	#16b	Report category boundaries when continuous variables were categorized	8-16
Main results	#16c	If relevant, consider translating estimates of relative risk into absolute risk for a meaningful time period	16
Other analyses	#17	Report other analyses done—e.g., analyses of subgroups and interactions, and sensitivity analyses	17
**Discussion**			
Key results	#18	Summarise key results with reference to study objectives	17
Limitations	#19	Discuss limitations of the study, taking into account sources of potential bias or imprecision. Discuss both direction and magnitude of any potential bias.	19

For peer review only

Interpretation	#20	Give a cautious overall interpretation considering objectives, limitations, multiplicity of analyses, results from similar studies, and other relevant evidence.	18, 20
Generalisability	#21	Discuss the generalisability (external validity) of the study results	20

Other Information

Funding	#22	Give the source of funding and the role of the funders for the present study and, if applicable, for the original study on which the present article is based	21

None The STROBE checklist is distributed under the terms of the Creative Commons Attribution License CC-BY. This checklist can be completed online using https://www.goodreports.org/, a tool made by the EQUATOR Network in collaboration with Penelope.ai

For peer review only

Trajectories of self-rated health in an older general population and their determinants: The Lifelines Cohort Study

Journal:	*BMJ Open*
Manuscript ID	bmjopen-2019-035012.R1
Article Type:	Original research
Date Submitted by the Author:	23-Dec-2019

Complete List of Authors:	Feenstra, Marlies; University of Groningen; Universitair Medisch Centrum Groningen, Internal Medicine and Geriatrics<br>van Munster, Barbara; Universitair Medisch Centrum Groningen, Internal Medicine and Geriatrics; Gelre Ziekenhuizen, Geriatrics<br>Macneil-Vroomen , Janet; Amsterdam Universitair Medische Centra, Geriatrics ; Yale School of Medicine<br>De Rooij, Sophia; Universitair Medisch Centrum Groningen, Internal Medicine and Geriatrics; Medical Centre Twente<br>Smidt, Nynke; Universitair Medisch Centrum Groningen, Epidemiology; Universitair Medisch Centrum Groningen, Internal Medicine and Geriatrics

**Primary Subject Heading**:	Epidemiology
Secondary Subject Heading:	Public health
Keywords:	EPIDEMIOLOGY, PUBLIC HEALTH, Risk management < HEALTH SERVICES ADMINISTRATION & MANAGEMENT

SCHOLARONE™
Manuscripts

I, the Submitting Author has the right to grant and does grant on behalf of all authors of the Work (as defined in the below author licence), an exclusive licence and/or a non-exclusive licence for contributions from authors who are: i) UK Crown employees; ii) where BMJ has agreed a CC-BY licence shall apply, and/or iii) in accordance with the terms applicable for US Federal Government officers or employees acting as part of their official duties; on a worldwide, perpetual, irrevocable, royalty-free basis to BMJ Publishing Group Ltd ("BMJ") its licensees and where the relevant Journal is co-owned by BMJ to the co-owners of the Journal, to publish the Work in this journal and any other BMJ products and to exploit all rights, as set out in our licence.

The Submitting Author accepts and understands that any supply made under these terms is made by BMJ to the Submitting Author unless you are acting as an employee on behalf of your employer or a postgraduate student of an affiliated institution which is paying any applicable article publishing charge ("APC") for Open Access articles. Where the Submitting Author wishes to make the Work available on an Open Access basis (and intends to pay the relevant APC), the terms of reuse of such Open Access shall be governed by a Creative Commons licence – details of these licences and which Creative Commons licence will apply to this Work are set out in our licence referred to above.

Other than as permitted in any relevant BMJ Author's Self Archiving Policies, I confirm this Work has not been accepted for publication elsewhere, is not being considered for publication elsewhere and does not duplicate material already published. I confirm all authors consent to publication of this Work and authorise the granting of this licence.

For peer review only

Trajectories of self-rated health in an older general population and their determinants: The Lifelines Cohort Study

Marlies Feenstra[a*], Barbara C van Munster[a,b], Janet L MacNeil Vroomen[c,d], Sophia E de Rooij[a,e], Nynke Smidt[a,f]

[a] University of Groningen, University Medical Center Groningen, Department of Internal Medicine and Geriatrics, Groningen, The Netherlands

[b] Gelre Hospitals, Department of Geriatrics, Apeldoorn, The Netherlands

[c] Amsterdam University Medical Centers, Department of Internal Medicine, Section of Geriatrics, Amsterdam, The Netherlands

[d] Yale School of Medicine, Department of Internal Medicine, Section of Geriatrics, New Haven, The United States of America

[e] Medical School Twente, Medical Spectrum Twente, Enschede, The Netherlands

[f] University of Groningen, University Medical Center Groningen, Department of Epidemiology, Groningen, The Netherlands

[*]**Corresponding author**: Marlies Feenstra

University of Groningen, University Medical Center Groningen

Department of Internal Medicine and Geriatrics

PO Box 30001, 9700 RB Groningen, The Netherlands

Tel: (+31) 50 361 3219 E-mail: m.feenstra01@umcg.nl

For peer review only

For peer review only - http://bmjopen.bmj.com/site/about/guidelines.xhtml

ABSTRACT

Objectives:

Poor self-rated health (SRH) is a strong predictor of premature mortality in older adults. Trajectories of poor SRH are associated with multimorbidity and unhealthy behaviors. Whether trajectories of SRH are associated with deviating physiological markers is unclear. This study identified trajectories of self-rated health (SRH) and investigated the associations of trajectory membership with chronic diseases, health risk behaviors, and physiological markers in community-dwelling older adults.

Study design and setting:

Prospective general population cohort

Participants:

Trajectories of SRH over 5 years were identified using data of 11 600 participants aged 65 years and older of the Lifelines Cohort Study.

Outcome measures:

Trajectories of SRH were the main outcome. Covariates included demographics (age, gender, education), chronic diseases, health-risk behavior (physical activity, smoking, drinking), and physiological markers (BMI, cardiovascular function , lung function , glucose metabolism , hematological condition , endocrine function, renal function, liver function, and cognitive function).

Results:

Four stable trajectories were identified, including excellent (n = 607, 6%), good (n = 2111, 19%), moderate (n = 7677, 65%), and poor SRH (n = 1205, 10%). Being female (OR: 1.4; 95%CI: 1.0 - 1.9), low education (OR: 2.1; 95%CI: 1.5 - 3.0), one (OR: 10.4; 95%CI: 7.4 - 14.7) or multiple chronic diseases (OR: 37.8; 95%CI: 22.4 -71.8), smoking (OR: 1.8; 95% CI:

For peer review only - http://bmjopen.bmj.com/site/about/guidelines.xhtml

1.0 - 3.2), physical inactivity (OR: 3.1; 95%CI: 1.8 - 5.2), alcohol abstinence (OR: 2.2; 95%CI: 1.4 - 3.2), and deviating physiological markers (OR: 1.5; 95%CI: 1.1 - 2.0) increase the odds for a higher probability of poor SRH trajectory membership compared to excellent SRH trajectory membership.

Conclusion:

SRH of community-dwelling older adults is stable over time with the majority (65%) having moderate SRH. Older adults with higher probabilities of poor SRH often have unfavorable health status.

Key words:

Longitudinal; Trajectory; Aging; Biomarkers; Health risk behavior; Multimorbidity.

For peer review only

STRENGHTS AND LIMITATIONS OF THIS STUDY

- This study concerns the evaluation of physiological markers as a determinant of self-rated health trajectories.
- The study results are representative for Dutch community dwelling adults aged 65 years and older.
- Reverse causation could not be eliminated.
- The number of chronic conditions were based on self-report, this could have caused non-differential misclassification bias.

Word count:

Abstract: 290

Main text: 3131

Tables: 4

Figures: 1

Appendices: 4 (A - D)

For peer review only - http://bmjopen.bmj.com/site/about/guidelines.xhtml

BACKGROUND

Self-rated health (SRH) is known as an inclusive measure of global health and is often used as a supplement to objective clinical measures of physical health, such as presence of disease and disability (1,2). In older adults, poor SRH is an independent and strong predictor of premature mortality (3,4). However, evidence for factors associated with poor SHR are predominantly cross-sectional and longitudinal evidence is required. Analysis of latent clusters of individuals who follow a similar pattern of SRH over time, so called trajectory analysis, can be used to explore the course of SRH in time within a certain population (5). Few studies have studied SRH in community-dwelling older adults by trajectory analysis revealing various numbers of identified trajectories (6–8). Distinct trajectories of SRH varied from persistently good (6,7), persistently moderate (7,8), persistently poor (6,7), declining (6–8) to improving trajectories of SRH (6). People in declining SRH trajectories were differentiated at baseline by older age, lower education level, and an increased number of chronic conditions compared to people in consistently good SRH trajectories (6–8). However, in these studies, other measures of determinants health status, such as abnormalities in physiological markers, like blood pressure, thyroid hormone levels, and glycated hemoglobin were not evaluated. Such markers reflect cross-sectional clinical parameters of physiological processes (9). Abnormal physiological processes may indicate pre-clinical prodromal phases of underlying diseases which are suggested to play a role in burden of disease expressed by poor SRH evaluations in older adults (1,4,10,11). We hypothesize that multi-morbidity, health risk behaviors, and deviations in physiological markers are associated with trajectories that lead to poor SRH.

The aim of this study is to identify classes of self-rated health over five years in community-dwelling older adults and to investigate whether group membership of SRH trajectories is

For peer review only - http://bmjopen.bmj.com/site/about/guidelines.xhtml

associated with self-reported chronic diseases, health risk behaviors, and physiological markers.

METHODS

Study population

A subsample of the adult Lifelines Cohort Study was used, including participants aged 65 years or older at baseline (n = 12 685). A detailed description of the complete Lifelines cohort profile is described elsewhere (12).

Measurements

Primary outcome measure

Repeated measures of self-rated health were assessed at baseline, 1.5 years, 3 years, and 5 years after baseline measurement by means of a self-reported question 'how would you rate your health in general? (excellent, very good, good, fair, poor)' (13,14). The single item SRH question with five response options is a valid and reliable measure of general health status in older adults (15–17).

Covariates

Demographics included *age, sex*, and *education level* (low, less than primary through lower secondary; intermediate, upper secondary through post-secondary, non-tertiary; high, short cycle tertiary and higher (18,19)).

Chronic diseases were categorized (none, one, two or more) based on a participant's baseline report on presence of the most burdensome chronic diseases as forecasted for the next decades by (RIVM, 2017), including dementia, myocardial infarction, osteoarthritis, cerebrovascular accident (CVA), diabetes, chronic obstructive pulmonary disease (COPD), cancer, anxiety, and mood disorders.

For peer review only - http://bmjopen.bmj.com/site/about/guidelines.xhtml

Health risk behaviors included *physical activity* (≥5, 2-4, ≤2 days/week physically active for at least 30 minutes (20)), *smoking* (never, former, current smoker), *alcohol consumption* (abstainer, low risk, at risk (21)). Low risk drinking is defined as no more than three drinks per day for women and men respectively, and no more than seven drinks per week (22).

Physiological markers included: body mass index (BMI) as a marker of body composition (23,24); Systolic and diastolic blood pressure was interpreted with total cholesterol and high density lipoprotein (HDL) ratio as a marker of cardiovascular function (23); Forced expired volume in one second (FEV1) and the forced vital capacity (FVC) ratio was used as a marker of lung function (25,26); Glycated hemoglobin (HbA1c) as a marker of glucose metabolism (23,27); Total hemoglobin (Hb) as a marker of hematological condition (27), Thyroid Stimulating Hormone (TSH) and free thyroxine (fT4) were used as markers of endocrine function (28–30); Estimated glomerular filtration rate (eGFR) by using the Cockcroft Gauld formula was used as a marker of renal function (31–33); Hepatic Steatosis Index (HSI) was used as a marker of liver function (34,35); and the mini mental state examination score (MMSE) was used as a marker of cognitive function (23,36). A detailed description of physiological markers used and clinical cut-offs are presented in Appendix A Table A1. Based on clinical cut-offs, both *individual physiological markers* (normal, abnormal values) and a *sum score of abnormal physiological markers* were used in the analyses (<3 vs. ≥3 abnormal physiological markers).

Statistical analyses

Baseline characteristics of all participants and classified by SRH trajectory groups were expressed in median and interquartile range (IQR) for continuous variables and proportions and percentages for categorical variables. To identify distinct trajectories of self-rated health over five year, latent class analyses were performed by using Group Based Trajectory Modeling (GBTM) (37). The trajectory model was built by a stepwise approach:

For peer review only - http://bmjopen.bmj.com/site/about/guidelines.xhtml

Step 1: The basic model was build including the four repeated measures of SRH using a censored normal model. Two up to six trajectories were considered after which the optimal number of trajectories was selected using highest Bayesian Information Criterion (BIC) (38), and Bayes factor (39). After the optimal number of trajectories was determined, optimal trajectory shape was determined by varying the growth terms. Optimal trajectory shape was evaluated based on 1. the probability of a person belonging to the selected trajectory (>0.7), 2. the odds of correct classification (>5.0), 3. close correspondence between the estimate of group membership probability and the proportion of individuals classified to the group, and 4. reasonable narrow confidence intervals for the estimates of group membership probability (40). For the latter two no formal criteria for maximum deviation were available.

Step 2: Multivariable multinomial logistic regression analyses were performed to estimate associations between the probability of SRH trajectory group assignment (result of step 1) and covariates. Three theoretical models were investigated. *Model 1*: chronic diseases and health behaviors; *Model 2*: model 1 plus physiological markers; *Model 3*: model 1 plus the sum score of abnormal physiological markers. For all determinants, multicollinearity was checked using Pearson's correlations. Baseline age, sex, and level of education were included in all models. Model selection was based on lowest BIC, and Akaike's Information Criterion (AIC) (41).

Step 3: Trajectories of SRH were re-estimated by including the covariates of the selected model out of step 2. This last step allows to evaluate the influence of one covariate on the probability of belonging to each trajectory taking into account the uncertainty of posterior group membership probability that is introduced by trajectory analysis. Wald statistics were applied for testing the differences between covariates across trajectory groups.

Data of participants with missing data of SRH at all time points were excluded from all analyses (n=1085 (9%)). Participants with missing SRH data at three or less time points were

For peer review only - http://bmjopen.bmj.com/site/about/guidelines.xhtml

handled using maximum likelihood estimation. Maximum likelihood estimation uses all available information from observed data for constructing the likely values for missing data (Nagin, 2005). From step 2 onwards, participants who had missing data for baseline covariates were excluded from further analyses (n=3010 (26%)). The flow of participants from the initial to the analytic sample is presented in Appendix B Figure B1.

Sensitivity analyses were performed by: 1) rerunning basic trajectory analysis accounting for non-random attrition (dual trajectory modeling), and 2) using a composite score for chronic diseases without anxiety and mood disorders. For all analyses Stata Statistical Software release 14 was used (StataCorp. 2015. College Station, Texas, USA) with the Traj plug-in (42,43).

For peer review only

RESULTS

Study population characteristics

Of all 11 600 participants, median age at baseline was 69 years (range 65 to 93), and 47% were male. Of this sample, 34% reported one chronic disease at baseline, 13% reported multimorbidity (≥2 chronic diseases), 57% had one or two abnormal physiological markers, and 38% had three or more abnormal physiological markers (Table 1). Over five years of follow-up, 497 people died (4%), and 3721 (32%) were lost to follow-up. The 3010 (26%) participants who were excluded from the analysis in step 2 and 3 due to missing covariates measured at baseline were older, more often female, lower educated, and had relatively less self-reported chronic diseases, but more abnormal values of physiological markers compared to the participants retained in the analysis (completers) (Table 2). One of the reasons for these missing data was that participant with low cognitive abilities (mini mental state examination <26) had a shorter proxy interview, which was the case in 1261 (42%) of the excluded participants.

For peer review only - http://bmjopen.bmj.com/site/about/guidelines.xhtml

Table 1. Baseline characteristics of all participants aged 65 years and older and categorized by SRH trajectory group.

**Characteristic**	**All**	**1. Excellent**	**2. Good**	**3. Moderate**	**4. Poor**
**n**	**11 600**	**602**	**2111**	**7677**	**1205**
**Demographics**					
Age, median (IQR25;75)	69 (66; 73)	68 (66; 72)	69 (66; 72)	69 (66; 72)	70 (67; 74)
range (years)	65-95	65-90	65-92	65-93	65-90
missing	-	-	-	-	-
Sex, n (%) male	5484 (47)	344 (57)	1161 (55)	3523 (46)	456 (38)
missing	-	-	-	-	-
Highest level of education, n(%)					
low	6563 (57)	301 (50)	1006 (48)	4482 (58)	774 (64)
intermediate	2037 (18)	107 (18)	407 (19)	1345 (18)	178 (15)
high	2239 (19)	168 (28)	592 (28)	1319 (17)	160 (13)
missing	*761 (7)*	*26 (4)*	*106 (5)*	*531 (7)*	*93 (8)*
**Health status, n (%)**					
Self-rated health					
excellent	645 (6)	373 (62)	246 (12)	26 (<1)	-
very good	2290 (20)	155 (26)	1326 (63)	804 (10)	5 (<1)
good	6358 (55)	4 (<1)	344 (16)	5805 (76)	205 (17)
fair	979 (8)	-	-	275 (4)	704 (58)
poor	20 (<1)	-	-	-	20 (2)
missing	*1308 (11)*	*78 (13)*	*208 (10)*	*845 (11)*	*286 (24)*
Chronic diseases (self-reported)					
none	6076 (52)	468 (78)	1386 (66)	3871 (50)	351 (29)
one	3979 (34)	116 (19)	604 (29)	2793 (36)	466 (39)
≥ 2	1545 (13)	24 (4)	121 (6)	1013 (13)	388 (32)
missing	-	-	-	-	-
**Health behaviors, n (%)**					
Physical activity for at least 30 minutes					
≥ 5 days/week	6395 (55)	368 (61)	1330 (63)	4226 (55)	471 (39)
2-4 days/week	2481 (21)	109 (18)	396 (19)	1743 (23)	233 (19)
≤ 1 day/week	761 (7)	27 (5)	93 (4)	512 (7)	129 (11)
missing	*1963 (17)*	*98 (16)*	*292 (14)*	*1196 (16)*	*371 (31)*

For peer review only - http://bmjopen.bmj.com/site/about/guidelines.xhtml

Table 1. Continued

**Characteristic**	**All**	**1. Excellent**	**2. Good**	**3. Moderate**	**4. Poor**
**n**	**11 600**	**602**	**2111**	**7677**	**1205**
**Health behaviors, n (%)**					
Smoking status					
never smoker	4453 (38)	238 (40)	802 (38)	2981 (39)	432 (36)
former smoker	5937 (51)	314 (52)	1121 (53)	3890 (51)	612 (51)
current smoker	789 (7)	37 (6)	128 (6)	530 (7)	94 (8)
missing	*421 (4)*	*13 (2)*	*60 (3)*	*276 (4)*	*67 (6)*
Alcohol consumption					
abstainer	2123 (18)	78 (13)	258 (12)	1479 (19)	307 (25)
low risk	3931 (34)	198 (33)	742 (35)	2674 (35)	317 (26)
at risk	3958 (34)	238 (40)	863 (41)	2566 (33)	290 (24)
missing	*1588 (14)*	*88 (15)*	*248 (12)*	*958 (12)*	*291 (24)*
**Physiological markers[a], n (%)**					
BMI[b] in kg / m^2					
<23	1323 (11)	107 (18)	295 (14)	822 (11)	99 (8)
≥ 23 & < 30[$]	8002 (69)	436 (72)	1560 (74)	5317 (69)	689 (57)
≥ 30	2264 (20)	64 (11)	256 (12)	1533 (20)	411 (34)
Blood pressure in mm Hg					
SBP ≤ 140/ 160[c] & DBP < 90[$]	6888 (59)	367 (61)	1271 (60)	4511 (59)	739 (61)
SBP ≤ 140/ 160[c] & DBP ≥ 90	92 (<1)	3 (<1)	20 (1)	64 (1)	5 (<1)
SBP > 140/ 160[c] & DBP < 90	3822 (33)	194 (32)	670 (32)	2560 (33)	398 (33)
SBP > 140/ 160[c] & DBP ≥ 90	774 (7)	42 (7)	145 (7)	528 (7)	59 (5)
CHOL/ HDL ratio					
< 3.5	5561 (48)	310 (51)	1040 (49)	3663 (48)	548 (45)
3.5-4.9[$]	4540 (39)	220 (37)	820 (39)	3022 (39)	478 (40)
> 5	1345 (12)	68 (11)	227 (11)	895 (12)	155 (13)
FEV1/ FVC ratio					
≥ 70[$]	8860 (76)	473 (79)	1625 (77)	5862 (76)	900 (75)
< 70	2740 (24)	134 (22)	486 (23)	1815 (24)	305 (25)

For peer review only - http://bmjopen.bmj.com/site/about/guidelines.xhtml

Table 1. Continued

**Characteristic**	**All**	**1. Excellent**	**2. Good**	**3. Moderate**	**4. Poor**
**n**	**11 600**	**602**	**2111**	**7677**	**1205**
**Physiological markers, n (%)**					
HbA1C in mmol/ mol (% of total Hb)					
< 48 (< 6.5%)[$]	9208 (79)	523 (87)	1767 (84)	6072 (79)	846 (70)
48-52 (6.5 -7%)	424 (4)	7 (1)	43 (2)	288 (4)	86 (7)
53-64 (7-8%)	324 (3)	0 (0)	39 (2)	217 (3)	68 (6)
> 64 (> 8%)	88 (1)	2 (<1)	7 (<1)	57 (1)	22 (2)
Hb in g/ dl (mmol/ L)					
< 12.1 / 13.7 (< 7.5/ 8.5)[d$]	886 (8)	46 (8)	166 (8)	549 (7)	125 (10)
≥ 12.1 / 13.7 (≥ 7.5/ 8.5)[d]	10 545 (91)	552 (92)	1921 (91)	7018 (91)	1054 (87)
TSH in mIU/L & fT4 in pmol/L					
TSH: 0.5-4.0 & fT4: 11-19.5[$]	2204 (19)	99 (16)	413 (20)	1466 (19)	226 (19)
TSH > 4.0 & fT4 ≥ 11 or <11	427 (4)	24 (4)	61 (3)	292 (4)	50 (4)
TSH < 0.5 & fT4 ≥ 11	81 (1)	6 (1)	8 (<1)	59 (1)	8 (1)
eGFR[e] in ml/min/1.73m^2					
≥ 90[$]	3809 (33)	179 (30)	622 (29)	2568 (33)	440 (37)
60-89	6577 (57)	375 (62)	1285 (61)	4315 (56)	602 (50)
45-59	898 (8)	40 (7)	166 (8)	594 (8)	98 (8)
< 45	151 (1)	4 (1)	14 (1)	98 (1)	35 (3)
HSI					
≤ 36[$]	2255 (19)	128 (21)	471 (22)	1486 (19)	170 (14)
> 36	1502 (4)	46 (8)	188 (9)	1031 (13)	237 (20)
MMSE score[f]					
25-30[$]	10738 (93)	552 (92)	1980 (94)	7178 (94)	1028 (85)
< 25	786 (7)	53 (9)	122 (6)	449 (6)	162 (14)
Sum score physiological markers					
none affected	600 (5)	33 (5)	132 (6)	386 (5)	49 (4)
≤ 2	6606 (57)	369 (61)	1298 (61)	4385 (57)	554 (46)
≥ 3	4394 (38)	202 (33)	670 (32)	2874 (37)	589 (49)

Notes: Percentages may not add up to 100% due to rounding.

[a.] Missing percentages for all physiological markers were <1% except for FEV1/FVC ratio (31%); TSH and fT4 (75%); and HSI (68%). Blood based markers are reported

For peer review only - http://bmjopen.bmj.com/site/about/guidelines.xhtml

in the International System of Units (SI) followed by conventional units if used in database. Values marked with $ are cut offs used to define normal values.
[b.] Cut-off was adjusted for age.
[c.] Higher cutoff for SBP was used if participants were aged ≥80.
[d.] Cut offs are adjusted for sex, men had higher cut-off.
[e.] Calculated by the Cockcroft Gauld formula using serum creatinin in umol/l, age, weight, and adjusted for sex.
[f.] Cut-offs are adjusted for level of education.
Abbreviations: BMI, body mass index; SBP, systolic blood pressure; DBP, diastolic blood pressure; CHOL, cholesterol; HDL, high-density lipoprotein; FEV1, forced expiration volume in 1 second; FVC, forced vital capacity; HbA1C, Hemoglobin A1C; Hb, hemoglobin; TSH, thyroid stimulating hormone; fT4, free thyroxine; eGFR, estimated glomeration filtration ratio; HSI, Hepatic Steatosis Index; MMSE, Mini Mental State Examination.

For peer review only

For peer review only - http://bmjopen.bmj.com/site/about/guidelines.xhtml

Table 2: Differences between completers and non-completers for baseline variables used in final model.

Characteristic	Completers N = 8590	Non-completers N = 3010	p-value
**Demographics**			
Age in years, median (IQR 25 - 75)[1]	68 (66 - 72)	69 (67 - 73)	<0.001
Male sex, n (%)[2]	4132 (48.1)	1352 (44.9)	0.001
Education, n (%)[2]			
low	4955 (57.7)	1608 (53.4)	<0.001
intermediate	1678 (19.5)	359 (11.9)	<0.001
high	1957 (22.8)	282 (9.4)	<0.001
missing percentage	0.5%	26%	
**Health status**			
Self-rated health, n (%)			
excellent	551 (6.4)	94 (3.1)	<0.001
very good	1982 (23.1)	308 (10.2)	<0.001
good	5274 (61.4)	1084 (36.0)	<0.001
fair	765 (8.9)	214 (7.1)	0.001
poor	16 (0.2)	4 (0.1)	0.129
missing percentage	0%	43%	
Self-reported chronic diseases, n (%)[2]			
none	4435 (51.6)	1641 (54.5)	0.003
one	3023 (35.2)	956 (31.8)	0.004
≥ 2	1132 (13.2)	413 (13.7)	0.399
missing percentage	0%	0%	
**Health behaviors**			
Physical activity for at least 30 minutes, n (%)[2]			
≥ 5 days/ week	5732 (66.7)	663 (22.0)	<0.001
2-4 days/ week	2191 (25.5)	290 (9.6)	<0.001
≤ 1 day/ week	667 (7.8)	94 (3.1)	<0.001
missing percentage	0%	65%	
Smoking status, n (%)[2]			
never smoker	3349 (39.0)	1104 (36.7)	0.007
former smoker	4628 (53.9)	1309 (43.5)	<0.001
current smoker	613 (7.1)	176 (5.8)	0.007
missing percentage	0%	13%	
Alcohol consumption, n (%)[2]			
abstainer	1760 (20.5)	362 (12.0)	<0.001
low-risk alcohol consumption	4224 (49.2)	561 (18.6)	<0.001
at risk alcohol consumption	2606 (30.3)	497 (16.5)	<0.001
missing percentage	0%	43%	
**Physiological markers[2]**			
≤ 2 affected	5859 (68.2)	1185 (39.4)	<0.001
≥ 3 affected	2731 (31.8)	1604 (53.3)	<0.001
missing percentage	0%	7%	

1. Equality of distributions was tested using the Wilcoxon Ranked Sum Test.

2. Equality of proportions was tested using the two sample test of proportions.

Abbreviations: n, number of participants; IQR, inter quartile range.

For peer review only - http://bmjopen.bmj.com/site/about/guidelines.xhtml

Trajectories of SRH over 5 years

Of all evaluated models, four trajectories of SRH with different intercepts, and all slopes close to zero showed the best fit (fit statistics are presented in Appendix C Tables C1 and C2). The four trajectories were identified as excellent, good, moderate, and poor SRH including 607 (5.6%), 2111 (18.8%), 7677 (65.3%), and 1205 (9.6%) participants, respectively (Figure 1; Appendix C Figure C1).

Table 1 presents baseline characteristics of participants in all trajectory groups. People having the highest probability of poor SRH trajectory membership were on average older, more often female, lower educated, more often physically inactive, more often alcohol abstainer, and they had more self-reported chronic diseases compared to people who have highest probabilities of assignment to the excellent, good and moderate SRH trajectories. Concerning objectively measured physiological markers, people having the highest probability of poor SRH trajectory membership had higher BMI, less often high blood pressure, but more often high CHOL/HDL ratio, higher Hb levels, higher HSI index, and they scored lower on cognitive function compared to people with highest probability of assignment to moderate, good and excellent SRH trajectories. In addition, people with the highest probability for poor SRH trajectory membership had more abnormal values of physiological markers compared to people with highest probability of assignment to moderate, good and excellent SRH trajectories.

(Figure 1 here)

Identification of covariates of trajectory membership probability

Table 3 presents the results from multivariate logistic regression analyses on probability of group membership of SRH. Model 2 performed worse compared to model 1 (BIC: -61 942; AIC:1.811). The simplest model with only self-reported covariates (model 1) had lowest BIC (-62 488), but higher AIC (1.807) compared to model 3 that included a sum score of physiological markers as well (BIC:-61 718; AIC: 1.804).

For peer review only - http://bmjopen.bmj.com/site/about/guidelines.xhtml

Table 3. Regression estimates (relative risk ratios and 95% confidence intervals) of poor SRH relative to excellent SRH from multivariate logistic regression models on SRH trajectory group membership.

Covariate	Excellent SRH	Poor SRH trajectory Model 1[a] n = 8679	Model 2[a] n = 8679	Model 3[a] n = 8590
Age	Ref.	1.01 (0.99; 1.04)	1.02 (0.99; 1.05)	1.01 (0.98; 1.04)
Sex,				
male	Ref.	Ref.	Ref.	Ref.
female	Ref.	1.44 (1.09; 1.90)	1.66 (1.24; 2.22)	1.46 (1.10; 1.94)
Education				
low	Ref.	Ref.	Ref.	Ref.
intermediate	Ref.	0.76 (0.55; 1.05)	0.77 (0.56 ; 1.07)	0.79 (0.57; 1.10)
high	Ref.	0.50 (0.37; 0.68)	0.56 (0.42; 0.77)	0.54 (0.40; 0.74)
Chronic diseases				
none	Ref.	Ref.	Ref.	Ref.
one	Ref.	7.80 (5.74; 10.61)	7.03 (5.16; 9.57)	7.76 (5.70; 10.58)
≥ 2	Ref.	26.42 (16.12; 43.30)	21.11 (12.80; 34.82)	25.08 (15.28; 41.17)
Physical activity for at least 30 minutes				
≥ 5 days/ week	Ref.	Ref.	Ref.	Ref.
2-4 days/ week	Ref.	1.63 (1.22; 2.18)	1.55 (1.16 ; 2.08)	1.61 (1.20; 2.15)
≤ 1 day/ week	Ref.	2.82 (1.75; 4.54)	2.55 (1.58 ; 4.13)	2.85 (1.76; 4.59)
Smoking status				
never	Ref.	Ref.	Ref.	Ref.
former	Ref.	1.40 (1.07; 1.83)	1.38 (1.05 ; 1.80)	1.39 (1.06; 1.82)
current	Ref.	1.71 (1.03; 2.85)	1.70 (1.01 ; 2.84)	1.65 (0.98; 2.78)
Alcohol consumption				
abstainer	Ref.	Ref.	Ref.	Ref.
low risk	Ref.	0.51 (0.36; 0.71)	0.53 (0.38; 0.75)	0.50 (0.35; 0.71)
at risk	Ref.	0.48 (0.33; 0.69)	0.51 (0.35; 0.74)	0.47 (0.33; 0.69)
Abnormal values of physiological markers[b]				
body composition	Ref.		1.35 (1.03; 1.76)	
cardiovascular function	Ref.		1.36 (1.06; 1.73)	
lung function	Ref.		1.12 (0.84; 1.50)	
glucose metabolism	Ref.		3.77 (1.71; 8.31)	
hematological cond.	Ref.		1.48 (0.95; 2.31)	
endocrine function	Ref.		0.97 (0.53; 1.79)	
renal function	Ref.		0.74 (0.56; 0.97)	
liver function	Ref.		1.78 (1.16; 2.74)	
cognitive function	Ref.		1.53 (1.00; 2.34)	
Sum score of physiological markers				
≤ 2 affected	Ref.			Ref.
≥ 3 affected	Ref.			1.51 (1.16; 1.96)

Notes: Participants with missing data for covariates were excluded from the analyses.

[a.] Fit statistics: Model 1: AIC: 1.807, BIC: -62488; Model 2: AIC: 1.811, BIC:-61942; Model 3:AIC:1.804, BIC:-61718.

[b.] Participants with normal values of the physiological markers were used as the reference category.

Abbreviations: ref, reference category; n, number of participants; AIC, Akaike Information Criterium; BIC, Bayesian Information Criterion.

For peer review only - http://bmjopen.bmj.com/site/about/guidelines.xhtml

However, both models had different sample sizes due to missing values for physiological markers in model 3. Taking into account the exploratory nature of this step in the analysis, type II error (an underfit model) would be more undesirable than type I error (an overfit model). Therefore the covariates included in model 3 were used for the final model (see Table 3, model 3).

Final model adjusted for associated covariates

The final trajectory model was modeled by jointly estimating the basic model and the covariates age, sex, educational level, self-reported chronic diseases, physical activity behavior, smoking behavior, alcohol consumption, and the sum score of affected physiological markers as risk factors. The final model assigned 471 (5.5%), 1716 (20.0%), 5637 (65.6%), and 766 (8.9%) people to the excellent, good, moderate, and poor SRH trajectories. The final model including covariates showed best fit statistics of posterior probability of group assignment (Appendix D, Table D1). The basic model overrepresented the proportion of participants with highest probability of poor and moderate SRH trajectory membership, and underrepresented the proportion of people with highest probability of excellent and good trajectory membership, compared to the final model that took into account the effect of covariates (Appendix D, Table D1).

Table 4 presents the odds ratios of each of the evaluated covariates of people with highest probability of poor, moderate, and good SRH trajectory membership using the excellent SRH trajectory as reference category. Increasing number of chronic diseases increased the odds of higher probability of poor SRH trajectory membership relative to the probability of excellent SRH trajectory membership (OR: 10.38; 95% CI: 7.38 - 14.72 for one chronic disease, OR: 37.79; 95% CI 22.35 - 71.75 for two or more chronic diseases). Female gender, low education level, physical inactivity, (former) smoking, alcohol abstinence, and presence of 3 or more abnormal values of physiological markers increased the odds of the probability of poor SRH

For peer review only - http://bmjopen.bmj.com/site/about/guidelines.xhtml

trajectory membership relative to the probability of excellent SRH trajectory membership (Table 4).

For peer review only

For peer review only - http://bmjopen.bmj.com/site/about/guidelines.xhtml

Table 4. Odds ratios and 95% confidence intervals per predictor for being member of the good, moderate or poor SRH trajectory group relative to the excellent group (n = 8590[a]).

Predictor	Odds ratios (95% Confidence Interval)			
	**Exc. SRH n = 471**	**Good SRH n = 1716**	**Moderate SRH n = 5637**	**Poor SRH n = 766**
Age				
65-69	Ref.	Ref.	Ref.	Ref.
70-74	Ref.	0.99 (0.75; 1.33)	0.93 (0.72; 1.19)	1.03 (0.77; 1.41)
75-79	Ref.	1.38 (0.89; 2.39)	1.33 (0.88; 2.18)	1.34 (0.81; 2.30)
≥ 80	Ref.	1.15 (0.56; 2.59)	1.08 (0.60; 2.31)	1.12 (0.56; 2.78)
Sex,				
male	Ref.	Ref.	Ref.	Ref.
female$	Ref.	1.03 (0.76; 1.39)	1.21 (0.95; 1.55)	1.43 (1.03; 1.94)
Education				
low	Ref.	Ref.	Ref.	Ref.
intermediate$	Ref.	1.10 (0.78; 1.53)	0.87 (0.646; 1.19)	0.76 (0.51; 1.12)
high$	Ref.	0.96 (0.73; 1.28)	0.54 (0.41; 0.68)	0.47 (0.33; 0.66)
Chronic diseases				
none	Ref.	Ref.	Ref.	Ref.
one	Ref.	2.11 (1.54; 2.93)	3.55 (2.80; 4.94)	10.38 (7.38; 14.72)
≥ 2	Ref.	1.60 (0.92; 3.30)	5.29 (3.35; 10.52)	37.79 (22.35; 71.75)
Physical activity for at least 30 minutes				
≥ 5 days/ week	Ref.	Ref.	Ref.	Ref.
2-4 days/ week$	Ref.	0.99 (0.76; 1.39)	1.35 (1.08; 1.80)	1.61 (1.18; 2.20)
≤ 1 day/ week	Ref.	0.95 (0.54; 1.76)	1.42 (0.90; 2.40)	3.12 (1.76; 5.16)
Smoking status				
never	Ref.	Ref.	Ref.	Ref.
former#	Ref.	1.08 (0.82; 1.42)	1.15 (0.91; 1.44)	1.48 (1.11; 1.98)
current$	Ref.	1.09 (0.66; 1.95)	1.42 (0.93; 2.30)	1.80 (1.02; 3.16)
Alcohol consumption				
abstainer	Ref.	Ref.	Ref.	Ref.
low risk	Ref.	1.38 (0.93; 2.16)	0.86 (0.62; 1.19)	0.52 (0.35; 0.77)
at risk	Ref.	1.40 (0.97; 2.12)	0.78 (0.57; 1.10)	0.46 (0.31; 0.70)
Sum score of physiological markers				
≤ 2 affected	Ref.	Ref.	Ref.	Ref.
≥ 3 affected	Ref.	0.89 (0.69; 1.21)	1.10 (0.88; 1.45)	1.50 (1.14; 2.03)

Final trajectory model including identified predictors of SRH trajectory membership by multinomial logistic regression analysis (table 2, model 3) adjusted for age (5 year intervals from 65 years old), education, and sex.

[a.] 3010 of 11.600 participants aged 65 years and older were excluded from the analysis due to missing data on covariates included in the final model.

$ Wald tests showed no differences between poor and moderate SRH trajectories

Wald tests showed no differences between moderate and good SRH trajectories

Abbreviations: Exc., excellent; Ref., reference category; SRH, self-rated health.

For peer review only - http://bmjopen.bmj.com/site/about/guidelines.xhtml

Wald tests implied that all trajectory groups were distinguished by the number of self-reported chronic diseases, alcohol consumption, and the sum score of affected physiological markers (p-values <0.001). However, the results presented in Table 4 should be interpreted with caution as all OR calculations are affected by the covariates that were included in the multinomial model to determine the probability of SRH trajectory membership.

Sensitivity analysis including alteration of the composite measure for multimorbidity without anxiety and depressive disorders did not alter trajectory group sizes, shapes, and odds ratios (results not shown). Dual trajectory modeling accounting for non-random attrition showed constant annual attrition probabilities between 10% (good SRH) and 17% (poor SRH) for all trajectory groups (Appendix D, Figure D1). Posterior probability of group assignment did not improve when modeling the trajectories accounting for attrition bias (Appendix D, Table D1).

DISCUSSION

In this sample of an ongoing large cohort study of Dutch community-dwelling older adults, four stable trajectories of SRH over five years were identified. The majority (65.3%) of the participants were classified into the moderate SRH category, followed by good (18.8%), poor (10.2%), and excellent (5.6%) SRH. The results of our study confirmed our a priori hypothesis that the probability of poor SRH trajectory membership was associated with multimorbidity, health risk behaviors, and abnormalities in physiological markers. The number of chronic diseases seems to be one of the key factors that determines someone's probability of SRH trajectory membership, as this was the only covariate under consideration that was significantly associated in all SRH trajectories. In addition, the probability of poor SRH trajectory membership was associated with being female, a low education level, health risk behaviors, and presence of three or more affected physiological markers.

For peer review only - http://bmjopen.bmj.com/site/about/guidelines.xhtml

Contrary to previous studies investigating trajectories of SRH, this study identified only stable trajectories of self-rated health of older community-dwelling adults during five years (6–8,44). Other studies with comparable measurement intervals, and study duration identified the majority of their participants in the stable trajectories as well, however they also identified small groups with declining and improving trajectories (6,8). Sample size was not the limiting factor to identify more groups, however, the posterior diagnostic criteria became worse when adding more than four trajectory groups, indicating four groups was the optimum for our sample. Participants of the current study were older than the populations used in other studies investigating trajectories of SRH. Response shift in SRH is known to occur among older adults (45). Compared to their younger counterparts, older adults are suggested to base their SRH more on psychological and life-style behaviors, and less on functional status and physical health, which might indicate reprioritization response shift (46,47). Furthermore, older adults adapt their standards of good health over time, also known as recalibration response shift (45). In addition, cognitive strategies to accept negative outcomes, as well as someone's beliefs contribute to enhanced levels of wellbeing, despite negative health outcomes (48), which can explain the stable trajectories of SRH over time in the present study sample.

Consistent with other studies investigating trajectories of SRH, we found strong associations between increasing numbers of baseline self-reported chronic diseases and the probability of poor SRH trajectory membership (6–8). When participants reported only one chronic disease, they had a two, three-and-half, and ten times higher odds of being a member of the good, moderate, and poor SRH trajectory compared to the probability of excellent SRH trajectory membership, respectively. People suffering two or more self-reported chronic diseases were 38 times more likely for having a higher probability for poor SRH trajectory membership

For peer review only - http://bmjopen.bmj.com/site/about/guidelines.xhtml

rather than a high probability for excellent SRH trajectory membership. Earlier studies found weaker associations between the probability of poor SRH trajectory membership and the number of chronic diseases (7,8). The difference in results might be explained by the different number and combinations of covariates used as predictors in different studies. For instance, previous studies focused on chronic physical health disorders to calculate a composite measure of multimorbidity (6,7). For this study, the eleven most burdensome chronic diseases forecasted for the next decades by the Dutch National Institute for Public Health and the Environment were used to measure chronic diseases, which included depression and anxiety disorders. The inclusion of depression and anxiety disorders in our composite measure of chronic diseases may have led to the strong associations between self-rated chronic diseases and the probability of poor SRH trajectory membership in the present study, because depressive symptoms are considered a risk factor for poor SRH (49). However, sensitivity analyses excluding depression and anxiety disorders in the composite score for chronic diseases led to similar results. Therefore, it is not expected that the differences in composite measures for chronic diseases explain the differences in magnitude of odds for the probability of poor SRH trajectory membership with increasing number of chronic diseases found in the present study compared to previous studies.

Strengths of this study are the large sample size, and short measurement intervals for SRH that contribute to the robustness of the findings. In addition, the use of physiological markers next to self-reported data was, to the best of our knowledge, not previously investigated in combination with trajectory analyses. There were limitations as well. Firstly, although we found a strong association between self-reported diseases and higher probability of poor SRH trajectory membership, we cannot rule out reverse causation. The presented odds ratios only measure relative change on group level and are not suited to generalize to individual

For peer review only - http://bmjopen.bmj.com/site/about/guidelines.xhtml

probability of group membership. It is therefore hard to translate these results into concrete clinical implications, as there will always be people having multimorbidity combined with excellent self-rated health. Second, in this older population, the use of self-reported measurements used for measuring the number of chronic diseases may have led to an over- or underestimation of the prevalence of diseases due to non-differential misclassification bias. Finally, attrition may have threatened the generalizability of our results (50). However, sensitivity analysis with trajectories jointly modeled with attrition (51) did not improve group allocation probabilities. In addition, constant annual attrition probabilities below 20% for all groups were identified, which led us to conclude that attrition rates were constant among all trajectory groups.

IMPLICATIONS AND CONCLUSIONS

The present study identified four stable trajectories of SRH over five years in Dutch community-dwelling, older adults where the majority of the sample had moderate SRH. Being female, lower levels of education, health risk behaviors (smoking, physical inactivity, and alcohol abstinence), and presence of three or more abnormal physiological markers were associated with higher probability of poor SRH trajectory membership. The identified modifiable determinants may provide a basis for future preventive strategies.

For peer review only - http://bmjopen.bmj.com/site/about/guidelines.xhtml

DECLARATIONS

Ethical considerations

The Lifelines Cohort study was approved by the research ethics committee of the University Medical Center Groningen, The Netherlands (registration number: 2007/152). All participants provided written informed consent before study enrollment.

Data Sharing Statement

The Lifelines facility is open for all researchers. Information on the application procedure for data access is described on www.lifelines.nl. Researchers interested in queries related to data access may contact the Lifelines Research Office via data@lifelines.nl.

Funding

This work was supported by the University of Groningen, in collaboration with the University Medical Center of Groningen, departments of epidemiology and internal medicine and geriatrics. The Lifelines Biobank initiative was funded by Fonds Economische Structuurversterking (FES), Samenwerkingsverband Noord Nederland (SNN), and Ruimtelijk Economisch Programma (REP). JMV is funded through the Netherlands Organization for Health Research and Development (NWO-ZonMw), grant number 91619060.

Conflict of interest

None

Acknowledgements

The authors wish to acknowledge the service of the Lifelines Cohort Study and all study participants.

For peer review only - http://bmjopen.bmj.com/site/about/guidelines.xhtml

Patient and public involvement statement

This research as well as the Lifelines Cohort Study database development was performed without public or patient involvement.

Author contributions

NS obtained funding and supervised the project. MF performed statistical analyses and wrote the first draft of the manuscript. NS and JMV aided in interpreting the results. MF, BVM, JMV, SDR, and NS were involved in the study design, revising manuscript draft for important intellectual content, and gave approval for the final manuscript, and thereby taking full responsibility for the work and manuscript content.

For peer review only

For peer review only - http://bmjopen.bmj.com/site/about/guidelines.xhtml

REFERENCES

1. Jylhä M. What is self-rated health and why does it predict mortality? Towards a unified conceptual model. *Soc Sci Med* 2009;69(3):307–16.

2. Tissue T. Another look at self-rated health among the elderly. *J Gerontol* 1972;27(1):91–4.

3. DeSalvo KB, Bloser N, Reynolds K, et al. Mortality Prediction with a Single General Self-Rated Health Question A Meta-Analysis. *J Gen Intern Med* 2005;20:267–75.

4. Idler EL, Benyamini Y. Self-Rated Health and Mortality : A Review of Twenty-Seven Community Studies. *J Health Soc Behav* 1997;38(1):21–37.

5. Nagin DS, Odgers CL. Group-based trajectory modeling in clinical research. *Annu Rev Clin Psychol* 2010;6:109–38.

6. Schmitz N, Gariépy G, Smith KJ, et al. Trajectories of self-rated health in people with diabetes: Associations with functioning in a prospective community sample. *PLoS One* 2013;8(12):1–7.

7. Lee HL, Huang HC, Lee M Der, et al. Factors affecting trajectory patterns of self-rated health (SRH) in an older population-A community-based longitudinal study. *Arch Gerontol Geriatr* 2012;54(3):334–41.

8. Ayyagari P, Ullrich F, Malmstrom TK, et al. Self-Rated Health Trajectories in the African American Health Cohort. *PLoS One* 2012;7(12).

9. FDA-NIH Biomarker Working Group. BEST (Biomarkers, EndpointS, and other Tools) Resource. Silver Spring: Food and Drug Administration; Bethesda: National

For peer review only

Institutes of Health. 2016.

10. Stenholm S, Pentti J, Kawachi I, et al. Self-rated health in the last 12 years of life compared to matched surviving controls: The health and retirement study. *PLoS One* 2014;9(9): e107879.

11. Stenholm S, Kivimäki M, Jylhä M, et al. Trajectories of self-rated health in the last 15 years of life by cause of death. *Eur J Epidemiol* 2016;31(2):177–85.

12. Scholtens S, Smidt N, Swertz MA, et al. Cohort Profile: LifeLines, a three-generation cohort study and biobank. *Int J Epidemiol* 2015;44(4):1172–80.

13. Ware JE, Sherbourne CD. The MOS 36-item short-form health survey (SF-36). I. Conceptual framework and item selection. *Med Care* 1992 Jun;30(6):473–83.

14. Aaronson NK, Muller M, Cohen PD, et al. Translation, validation, and norming of the Dutch language version of the SF-36 Health Survey in community and chronic disease populations. *J Clin Epidemiol* 1998 Nov;51(11):1055–68.

15. Eriksson I, Undén A-L, Elofsson S. Research on self-rated health measurement scale. *Int J Epidemiol* 2001;30:326–33.

16. DeSalvo KB, Fisher WP, Tran K, Bloser N, Merrill W, Peabody J. Assessing measurement properties of two single-item general health measures. *Qual Life Res* 2006;15(2):191–201.

17. Cox B, van Oyen H, Cambois E, Jagger C, Roy S Le, Robine JM, et al. The reliability of the Minimum European Health Module. *Int J Public Health* 2009;54(2):55–60.

18. Unesco Institute for Statistics. International Standard Classification of Education

For peer review only

For peer review only - http://bmjopen.bmj.com/site/about/guidelines.xhtml

ISCED 2011. Montreal: UNESCO Institute for Statistics; 2012. 88p.

19. Centraal Bureau voor de Statistiek. SOI 2016, Standaard Onderwijsindeling 2016. Den Haag / Heerlen: CBS; 2017. 40p.

20. Kemper HCG, Ooijendijk WTM, Stiggelbout M. Consensus over de Nederlandse norm voor gezond bewegen. *Tijdschr Soc Gezondheidsz* 2000;78(3):180–3.

21. Gezondheidsraad. Richtlijnen goede voeding 2015. Den Haag: Gezondheidsraad; 2015. 94 p. Reportno.: 2015/24.

22. National Institute on Alcohol Abuse and Alcoholism. Rethinking Drinking Alcohol and your health. 15-3770. Bethesda MD; 2016. https://www.rethinkingdrinking.niaaa.nih.gov (accessed 10 December 2019)

23. Mathers J, Deary I, Kuh D, et al. Guidelines for biomarkers of healthy ageing. 93p. https://mrc.ukri.org/documents/pdf/biomarkers-of-healthy-ageing/ (accessed 4 October 2018)

24. Winter JE, Macinnis RJ, Wattanapenpaiboon N, et al. BMI and all-cause mortality in older adults: a meta-analysis. *Am J Clin Nutr* 2014;99:875–90.

25. Mannino DM, Diaz-Guzman E. Interpreting lung function data using 80% predicted and fixed thresholds identifies patients at increased risk of mortality. *Chest* 2012;141(1):73–80.

26. Sorino C, Sherrill D, Guerra S, et al. Prognostic value of FEV1/FEV6 in elderly people. *Clin Physiol Funct Imaging* 2011;31(2):101–7.

27. Fried LP, Xue QL, Cappola AR, et al. Nonlinear multisystem physiological

For peer review only

For peer review only - http://bmjopen.bmj.com/site/about/guidelines.xhtml

dysregulation associated with frailty in older women: Implications for etiology and treatment. *J Gerontol A Biol Sci Med Sci* 2009;64(10):1049–57.

28. Rodondi N, Den Elzen WPJ, Bauer DC, et al. Subclinical Hypothyroidism and the Risk of Coronary Heart Disease and Mortality. *JAMA* 2010;304(12):1365–74.

29. Pearce SHS, Razvi S, Yadegarfar ME, et al. Serum Thyroid Function, Mortality and Disability in Advanced Old Age: The Newcastle 85+ Study. *J Clin Endocrinol Metab* 2016;101(11):4385–94.

30. Mammen JS, McGready J, Ladenson PW, et al. Unstable Thyroid Function in Older Adults Is Caused by Alterations in Both Thyroid and Pituitary Physiology and Is Associated with Increased Mortality. *Thyroid* 2017;27(11):1370–77.

31. Montesanto A, De Rango F, Berardelli M, et al. Glomerular filtration rate in the elderly and in the oldest old: Correlation with frailty and mortality. *Age* 2014;36(3):1503–14.

32. Levey AS, Stevens LA, Schmid CH, et al. A new equation to estimate glomerular filtration rate. *Ann Intern Med* 2009;150(9):604–12.

33. Levey AS, Stevens LA. Estimating GFR using the CKD Epidemiology Collaboration (CKD-EPI) creatinine equation: more accurate GFR estimates, lower CKD prevalence estimates, and better risk predictions. *Am J Kidney Dis* 2010;55(4):622–7.

34. Kahl S, Strassburger K, Nowotny B, et al. Comparison of Liver Fat Indices for the Diagnosis of Hepatic Steatosis and Insulin Resistance. *PLoS One* 2014;9(4):e94059.

35. Lee J, Kim D, Jung H, et al. Hepatic steatosis index : A simple screening tool reflecting nonalcoholic fatty liver disease. *Dig Liver Dis* 2010;42(7):504–9.

For peer review only - http://bmjopen.bmj.com/site/about/guidelines.xhtml

36. Schmand B, Lindeboom J, Hooijer C, et al. Relation between education and dementia : the role of test bias revisited. *J Neurol Neurosurg psychiatry* 1995;59:170–4.

37. Nagin DS, Jones BL, Lima Passos V, et al. Group-based multi-trajectory modeling. *Stat Methods Med Res* 2016; 0(0):1-9.

38. Schwarz G. Estimating the dimension of a model. *Ann Stat* 1978;6(2):461–4.

39. Wasserman L. Bayesian Model Selection and Model Averaging. *J Math Psychol* 2000;44:92–107.

40. Nagin D. Group-based modeling of development. Cambridge, Massachusetts: Harvard University Press; 2005. 201 p.

41. Dziak JJ, Coffman DL, Lanza ST, et al. Sensitivity and specificity of information criteria. *Briefings in Bioinformatics* 2019; 00(0):1–13.

42. Jones BL, Nagin DS. A Stata plugin for estimating group-based trajectory models. Pittsburgh; 2012

43. Jones BL, Nagin DS. A Note on a Stata Plugin for Estimating Group-based Trajectory Models. *Sociol Methods Res* 2013;42(4):608–13.

44. Sacker A, Wiggins RD, Bartley M, et al. Self-rated health trajectories in the United States and the United Kingdom: A comparative study. *Am J Public Health* 2007;97(5):812–8.

45. Spuling SM, Wolff JK, Wurm S. Response shift in self-rated health after serious health events in old age. *Soc Sci Med* 2017;192:85–93.

46. Hoeymans N, Feskens EJM, Kromhout D, et al. Aging And The Relationship Between

For peer review only - http://bmjopen.bmj.com/site/about/guidelines.xhtml

Functional Status And Self-Rated Health In Elderly Men. *Soc Sci Med* 1997;45(10):1527–36.

47. Leinonen R, Heikkinen E, Jylhä M. Self-rated health and self-assessed change in health in elderly men and women - A five-year longitudinal study. *Soc Sci Med* 1998;46(4–5):591–7.

48. de Ridder D, Geenen R, Kuijer R, van Middendorp H. Psychological adjustment to chronic disease. *Lancet* 2008;372(9634):246–55.

49. Han B. Depressive Symptoms and Self-Rated Health in Community-Dwelling Older Adults: A Longitudinal Study. *J Am Geriatr Soc* 2002;50:1549–56.

50. Genbäck M, Ng N, Stanghellini E, et al. Predictors of decline in self ‑ reported health : addressing non ‑ ignorable dropout in longitudinal studies of aging. *Eur J Ageing* 2018;15(2):211–20.

51. Haviland AM, Jones BL, Nagin DS. Group-based Trajectory Modeling Extended to Account for Nonrandom Participant Attrition. *Sociol Methods Res* 2011;40(2):367–90.

For peer review only - http://bmjopen.bmj.com/site/about/guidelines.xhtml

FIGURE LEGEND

Figure 1. Non adjusted trajectories of SRH over five years using 11 600 adults aged 65 years and older of the Lifelines Cohort Study.

For peer review only

For peer review only - http://bmjopen.bmj.com/site/about/guidelines.xhtml

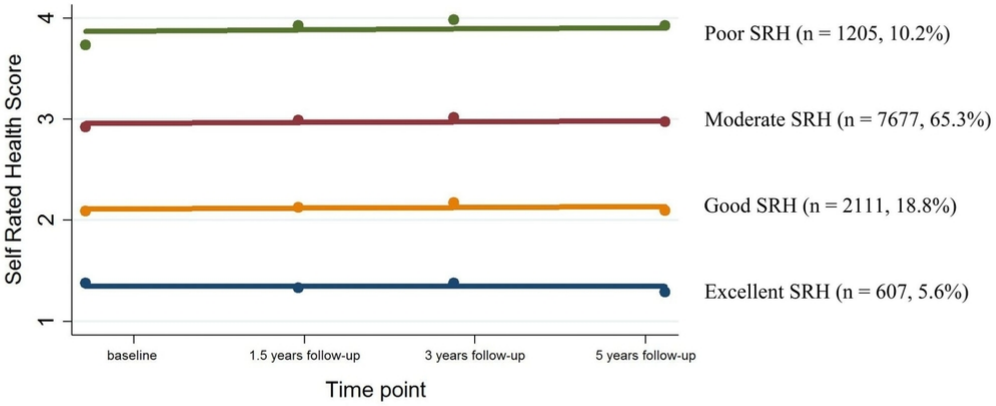

Figure 1: Non adjusted trajectories of SRH over five years using 11 600 adults aged 65 years and older of the Lifelines Cohort Study.

84x34mm (300 x 300 DPI)

APPENDIX A: PHYSIOLOGICAL MARKER SPECIFICATION

Table A1: Cut-offs used to define normal and affected values for markers per organsystem.

**Lung function**		
FEV1 (L) /FVC (L) ratio (multiplied by 100%)	Cut-off scores used (Quanjer et al., 2012):<br>Normal: ≥ 70%<br>Affected < 70%	
**Renal function**		
eGFR (in ml/min/1.73m2)	Estimated with the Cockcroft Gauld formula using serum creatine in umol/l (adjusted for age, sex, weight) (Cockcroft & Gault, 1976).<br>Cut-off scores used (Traynor, Mactier, Geddes, & Fox, 2006):<br>Normal: ≥90 ml/min/1.73m^2<br>Affected: <90 ml/min/1.73m^2	
**Endocrine function**[1]		
TSH (mIU/L)	Normscores (lab standards UMCG):<br>Low: <0.5 mIU/L<br>Normal: 0.5 – 4.0 mIU/L<br>High: ≥ 4.0 mIU/L	
fT4 (pmol/L)	Normscores (Boesten et al., 2012):<br>Low: < 11.0 pmol/L<br>Normal: 11.0 – 19.5 pmol/L<br>High: > 19.5 pmol/L	
**Immune function**		
Hb (mmol/L)	Different cut-offs used for men and women (lab standards UMCG).<br>Cut off used:<br>Men:<br>Normal: ≥8.5 and ≤ 11 mmol/L<br>Affected: <8.5, >11 mmol/L	 <br>Women:<br>Normal: ≥7.5 and ≤ 10 mmol/L<br>Affected: <7.5, >10 mmol/L
**Liver function**		
Hepatic Steatosis Index	Cut off used (Lee et al., 2010; Meems et al., 2015):<br>Normal: ≤ 36<br>Affected >36	
**Cognitive function**		
MMSE	Adjusted for level of education: primary education or less (max.6 years) and secondary or higher (>6 years) (Schmand, Lindeboom, Hooijer, & Jonker, 1995).<br>Cut-off used:<br>≤ Primary:<br>Normal: ≥25<br>Affected: <25	 <br><br>≥ Secondary:<br>Normal ≥27<br>Affected: <27
**Body composition**		
BMI (for Caucasian)	Age adjusted BMI cutoffs were used (Winter, Macinnis, Wattanapenpaiboon, & Nowson, 2014).<br>Cut offs used:<br>Normal: ≥23.0 BMI <30<br>Affected: <23 & BMI ≥30	
**Cardiovascular function**[2]		

For peer review only

SBP (mmHg)	Adjusted for age ((European Society of Hypertension/ European Society of Cardiology, 2014))<br>Cut-offs used:<br>Aged <80: Normal: ≤ 140 mmHg; High: >140 mmHg<br>Aged ≥80: Normal: ≤ 160 mmHg; High: >160 mmHg
DBP (mmHg)	Cut-off used (European Society of Hypertension/ European Society of Cardiology, 2014):<br>Normal: <90 mmHg<br>High: ≥90 mmHg
Total cholesterol (mmol/L)/ HDL (mmol/L) ratio	Cut offs used (European Society of Cardiology / European Atherosclerosis Society, 2016; Landelijke werkgroep Cardiovasculair risicomanagement, 2012):<br>Normal: <5.0<br>High ≥ 5.0
**Glucose metabolism**	
HbA1c (mmol (HbA1c) / mol (Hb))	Cut offs used (Fried et al., 2009):<br>Normal: < 48 mmol/mol (corresponding to 6,5% of total Hb)<br>Affected: ≥ 48 mmol/mol

Abbreviations: FER, forced expiratory ratio; eGFR, estimated glomerular filtration rate; TSH, thyroid stimulating hormone; FSH, follicle-stimulating hormone; HB, hemoglobin; MMSE, mini mental state examination; BMI, body mass index; SBP, systolic blood pressure; DBP, diastolic blood pressure; HDL, high density lipoprotein; HbA1C, glycated hemoglobin.

1. TSH cut-offs were interpreted with fT4; both TSH and fT4 should be in the normal range to score 'normal' concerning the endocrine system.

2. Blood pressure was interpreted with cholesterol levels; both diastolic and systolic bloodpressure and cholesterol/HDL ratio or should in the normal range to score 'normal' concerning the cardiovascular system.

References:

Boesten, L. S. M., Brugts, M. P., & van Rossum, A. P. (2012). Schildklierdiagnostiek : discrepantie FT4 en TSH. *Nederlands Tijdschrift Voor Geneeskunde*, *156*, A4167V.

Cockcroft, D. W., & Gault, H. (1976). Prediction of Creatinine Clearance from Serum Creatinine. *Nephron*, *16*(1), 31–41. European Society of Cardiology / European Atherosclerosis Society. (2016). 2016 ESC/EAS guidelines for the management of dyslipidemias. *European Heart Journal*, *37*, 2999–3058.

European Society of Hypertension/ European Society of Cardiology. (2014). 2014 guidelines for the management of arterial hypertension. *Primary Care Cardiovascular Journal*, *7*(2), 85–88.

Fried, L. P., Xue, Q. L., Cappola, A. R., Ferrucci, L., Chaves, P., Varadhan, R., … Bandeen-Roche, K. (2009). Nonlinear multisystem physiological dysregulation associated with frailty in older women: Implications for etiology and treatment. *Journals of Gerontology - Series A Biological Sciences and Medical Sciences*, *64*(10), 1049–1057.

Landelijke werkgroep Cardiovasculair risicomanagement. (2012). Dutch standard of cardiovasculair riskmanagement. *Huisarts Wet*, *55*(1), 14–28.

Lee, J., Kim, D., Jung, H., Lee, C., In, J., Kim, W., … Lee, H. (2010). Hepatic steatosis index : A simple screening tool reflecting nonalcoholic fatty liver disease. *Digestive and Liver Disease*,

For peer review only

42(7), 504–509.

Meems, L. M. G., De Borst, M. H., Postma, D. S., Vonk, J. M., Kremer, H. P. H., Schuttelaar, M. L. A., … De Boer, R. A. (2015). Low levels of Vitamin D are associated with multimorbidity: Results from the LifeLines Cohort Study. *Annals of Medicine*, *47*(6), 474–481.

Quanjer, P. H., Stanojevic, S., Cole, T. J., Baur, X., Hall, G. L., Culver, B. H., … Schindler, C. (2012). Multi-ethnic reference values for spirometry for the 3-95-yr age range: The global lung function 2012 equations. *European Respiratory Journal*, *40*(6), 1324–1343.

Schmand, B., Lindeboom, J., Hooijer, C., & Jonker, C. (1995). Relation between education and dementia : the role of test bias revisited. *Journal of Neurology, Neurosurgery and Psychiatry*, *59*, 170–174.

Traynor, J., Mactier, R., Geddes, C. C., & Fox, J. G. (2006). How to measure renal function in clinical practice: Estimated glomerular filtration rate in general practice. *Bmj*, *333*(7574), 918.2.

Winter, J. E., Macinnis, R. J., Wattanapenpaiboon, N., & Nowson, C. A. (2014). BMI and all-cause mortality in older adults: a meta-analysis. *Am J Clin Nutr*, *99*, 875–890.

For peer review only

For peer review only - http://bmjopen.bmj.com/site/about/guidelines.xhtml

APPENDIX B: FLOWCHART OF STUDY SAMPLE

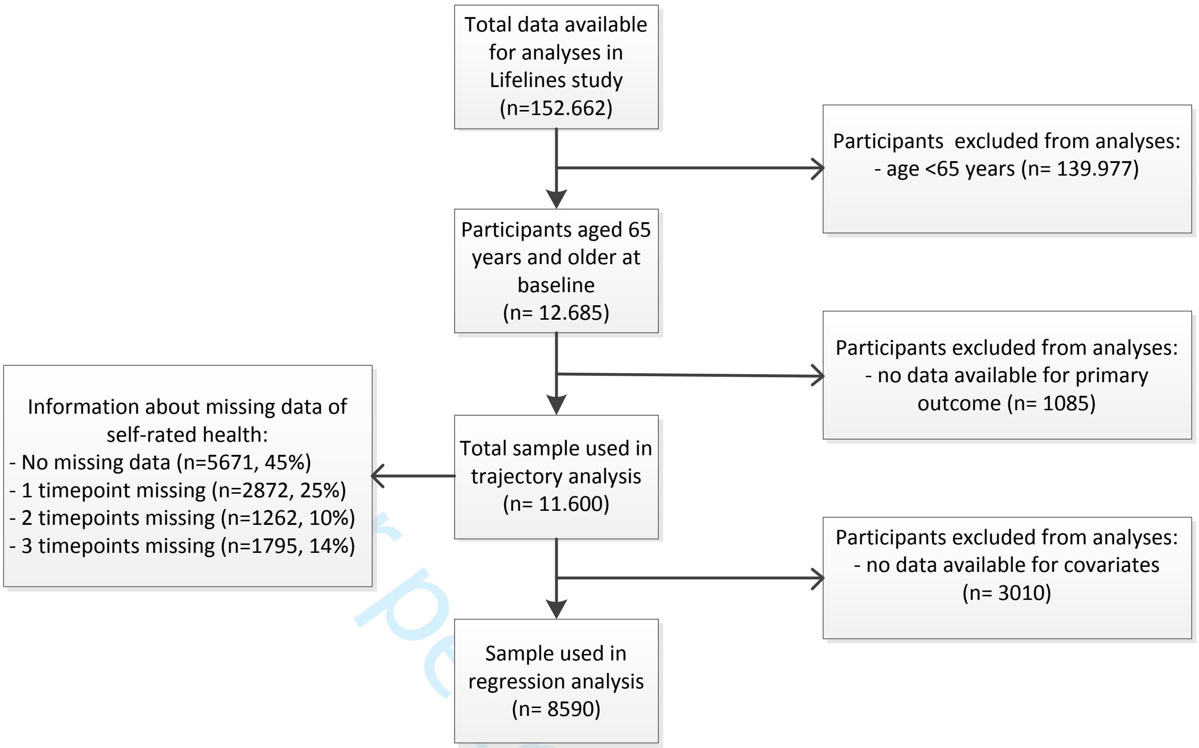

Figure B1. Flow of selection of study sample.

For peer review only - http://bmjopen.bmj.com/site/about/guidelines.xhtml

APPENDIX C: BASIC MODEL SPECIFICATIONS

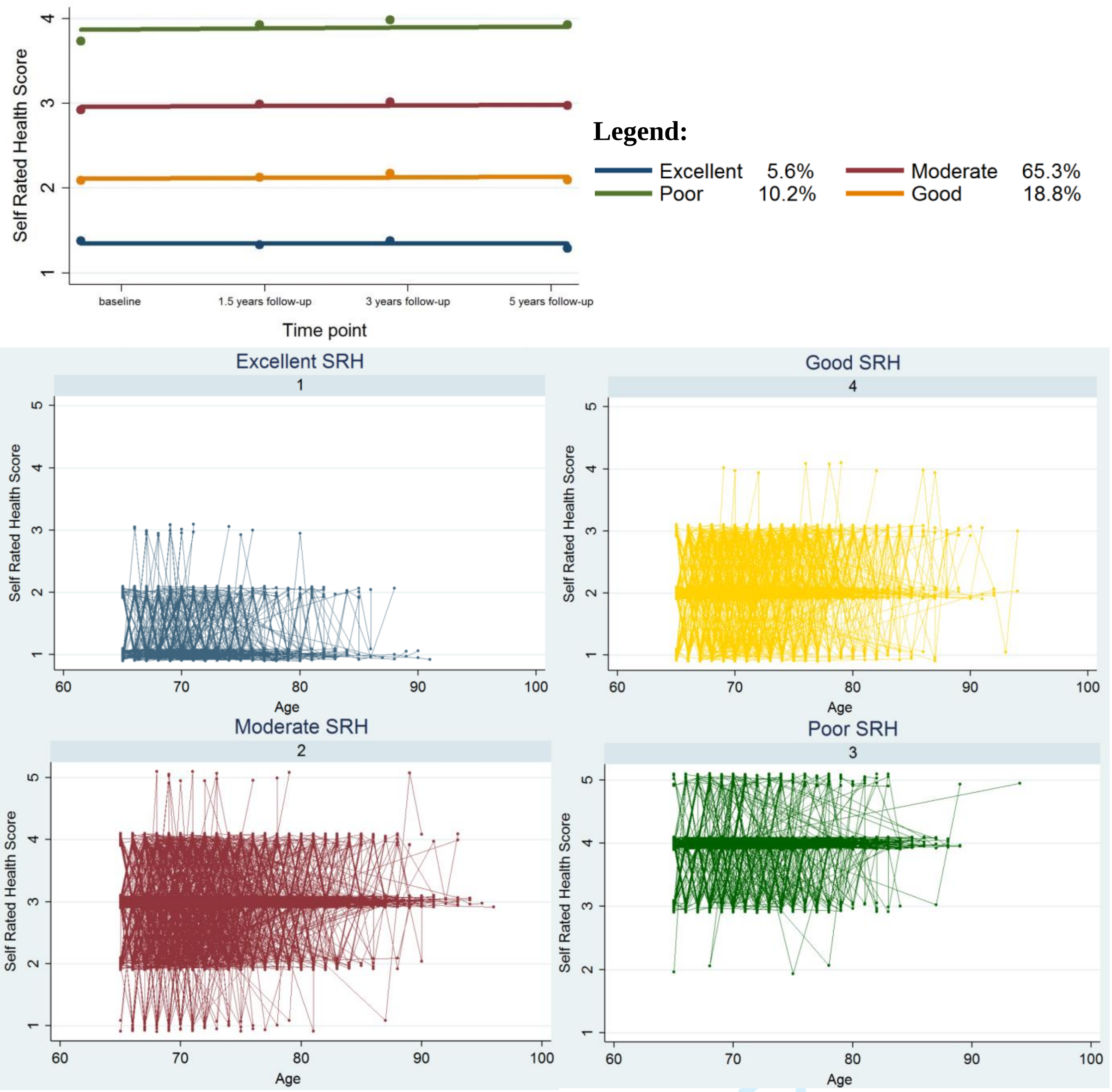

Figure C1: Basic mean trajectory groups of SRH (a), and observed individual trajectories per trajectory group (b-e) over five years of 11.600 people aged 65 years and older of the Lifelines Cohort. a. Dots represent the mean observed value per measurement moment; solid lines represent fit lines; dotted lines represent 95% confidence intervals of the fit lines.
b-e. Jittering was used for adding random noise to make all individual scores integer to avoid overlap of individual trajectories for people with identical trajectories.

For peer review only - http://bmjopen.bmj.com/site/about/guidelines.xhtml

Table C1: Bayesian Information Criteria and probability estimation Jeffreys's scale of evidence for Bayes factors of crude trajectory calculations with fixed quadratic growth terms used to select adequate number of groups.

**No. of groups**	**BIC** (n=11.600)	**Probability correct model**
2	-35016.79	0
3	-33001.46	0
4	-32240.77	0.98
5	-32259.49	< 0.01
6	-32278.20	< 0.01
7	-32244.54	0.02

Table C2: Posterior diagnostics of model performance of basic trajectory model.

**Group**	**Model estimate** ***(π^)***	**95% CI**	**Proportion classified** ***(p^)***	**Ave. PP**	**Odds correct classification**
1	.056	(.050; .062)	.052	.892	138.3
2	.653	(.642; .664)	.662	.941	8.4
3	.102	(.095; .109)	.104	.852	50.7
4	.188	(.179; .198)	.182	.863	27.1

For peer review only - http://bmjopen.bmj.com/site/about/guidelines.xhtml

APPENDIX D: SENSITIVITY ANALYSES

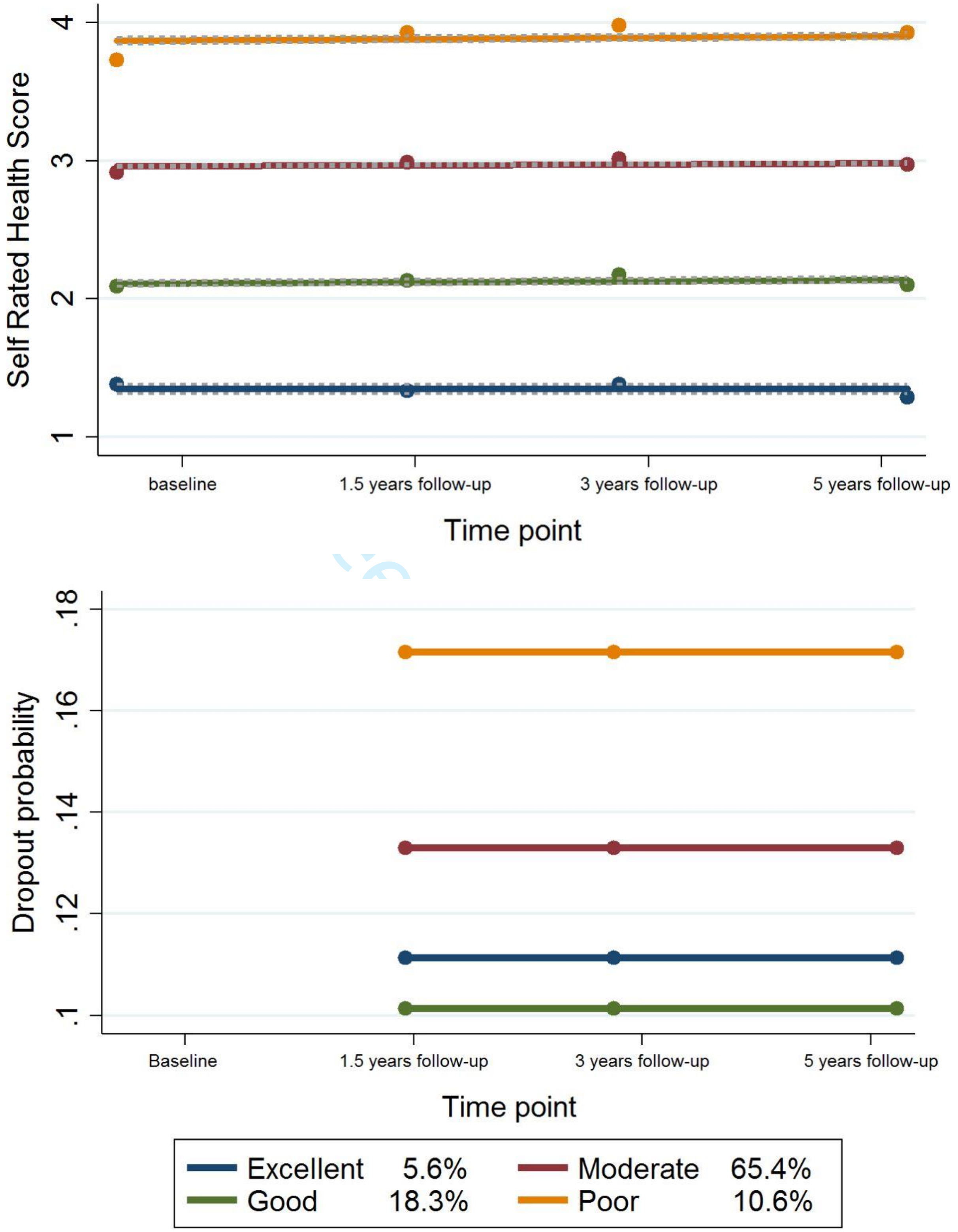

Figure D1: Trajectories of SRH jointly modelled with attrition. The upper plot represent trajectories of SRH accounted for attrition risk with probability for dropout per trajectory is presented in the lower plot. Dots represent the mean observed value per measurement moment; solid lines represent fit lines; dotted lines in the upper plot represent 95% confidence intervals of the fit lines.

For peer review only - http://bmjopen.bmj.com/site/about/guidelines.xhtml

Table D1. Comparison of posterior probability of assignment for the basic model, the model including covariates / risk factors, and the trajectory model that jointly modelled attrition (sensitivity analysis).

Group Allocation	N (%)	Excellent	Good	Moderate	Poor
**Basic model (step 1): posterior probability of assignment**					
Excellent	607 (5.6)	**0.89**	<0.01	0.01	0.05
Good	2111 (18.8)	0.11	**0.86**	0.06	<0.01
Moderate	8762 (65.3)	<0.01	0.15	**0.91**	0.08
Poor	1205 (10.2)	<0.01	<0.01	0.03	**0.85**
**Model with covariates (step 3): posterior probability of assignment**					
Excellent	471 (5.5)	**0.91**	0.05	<0.01	<0.01
Good	1716 (20.0)	0.09	**0.87**	0.04	<0.01
Moderate	5637 (65.6)	<0.01	0.08	**0.95**	0.09
Poor	766 (8.9)	<0.01	<0.01	0.02	**0.91**
**Model with attrition (sensitivity analysis): posterior probability of assignment**					
Excellent	609 (5.7)	**0.90**	0.05	0.01	<0.01
Good	2123 (18.7)	0.10	**0.86**	0.05	<0.01
Moderate	8762 (65.3)	<0.01	0.09	**0.91**	0.14
Poor	1191 (10.3)	<0.01	<0.01	0.04	**0.86**

*Rows may add to more than 1.0 due to rounding.

For peer review only

Reporting checklist for cohort study.

Based on the STROBE cohort guidelines.

Instructions to authors

Complete this checklist by entering the page numbers from your manuscript where readers will find each of the items listed below.

Your article may not currently address all the items on the checklist. Please modify your text to include the missing information. If you are certain that an item does not apply, please write "n/a" and provide a short explanation.

Upload your completed checklist as an extra file when you submit to a journal.

In your methods section, say that you used the STROBE cohortreporting guidelines, and cite them as:

von Elm E, Altman DG, Egger M, Pocock SJ, Gotzsche PC, Vandenbroucke JP. The Strengthening the Reporting of Observational Studies in Epidemiology (STROBE) Statement: guidelines for reporting observational studies.

For peer review only

		Reporting Item	Page Number
**Title and abstract**			
Title	#1a	Indicate the study’s design with a commonly used term in the title or the abstract	1
Abstract	#1b	Provide in the abstract an informative and balanced summary of what was done and what was found	2
**Introduction**			
Background / rationale	#2	Explain the scientific background and rationale for the investigation being reported	4
Objectives	#3	State specific objectives, including any prespecified hypotheses	4
**Methods**			
Study design	#4	Present key elements of study design early in the paper	5
Setting	#5	Describe the setting, locations, and relevant dates, including	5

		periods of recruitment, exposure, follow-up, and data collection	
Eligibility criteria	#6a	Give the eligibility criteria, and the sources and methods of selection of participants. Describe methods of follow-up.	5
Eligibility criteria	#6b	For matched studies, give matching criteria and number of exposed and unexposed	n/a, not matched
Variables	#7	Clearly define all outcomes, exposures, predictors, potential confounders, and effect modifiers. Give diagnostic criteria, if applicable	5-6
Data sources / measurement	#8	For each variable of interest give sources of data and details of methods of assessment (measurement). Describe comparability of assessment methods if there is more than one group. Give information separately for for exposed and unexposed groups if applicable.	Appendix A1
Bias	#9	Describe any efforts to address potential sources of bias	8
Study size	#10	Explain how the study size was arrived at	9
Quantitative variables	#11	Explain how quantitative variables were handled in the analyses. If applicable, describe which groupings were chosen, and why	6-7
Statistical methods	#12a	Describe all statistical methods, including those used to control for confounding	6-8
Statistical methods	#12b	Describe any methods used to examine subgroups and interactions	6-8
Statistical methods	#12c	Explain how missing data were addressed	8
Statistical methods	#12d	If applicable, explain how loss to follow-up was addressed	8
Statistical methods	#12e	Describe any sensitivity analyses	8
**Results**			
Participants	#13a	Report numbers of individuals at each stage of study—eg numbers potentially eligible, examined for eligibility,	8

For peer review only

		confirmed eligible, included in the study, completing follow-up, and analysed. Give information separately for for exposed and unexposed groups if applicable.	
Participants	#13b	Give reasons for non-participation at each stage	Appendix B
Participants	#13c	Consider use of a flow diagram	Appendix B
Descriptive data	#14a	Give characteristics of study participants (eg demographic, clinical, social) and information on exposures and potential confounders. Give information separately for exposed and unexposed groups if applicable.	8, 12
Descriptive data	#14b	Indicate number of participants with missing data for each variable of interest	9-11
Descriptive data	#14c	Summarise follow-up time (eg, average and total amount)	8
Outcome data	#15	Report numbers of outcome events or summary measures over time. Give information separately for exposed and unexposed groups if applicable.	9-11
Main results	#16a	Give unadjusted estimates and, if applicable, confounder-adjusted estimates and their precision (eg, 95% confidence interval). Make clear which confounders were adjusted for and why they were included	14-16
Main results	#16b	Report category boundaries when continuous variables were categorized	8-16
Main results	#16c	If relevant, consider translating estimates of relative risk into absolute risk for a meaningful time period	16
Other analyses	#17	Report other analyses done—e.g., analyses of subgroups and interactions, and sensitivity analyses	17
**Discussion**			
Key results	#18	Summarise key results with reference to study objectives	17
Limitations	#19	Discuss limitations of the study, taking into account sources of potential bias or imprecision. Discuss both direction and magnitude of any potential bias.	19

For peer review only

Interpretation	#20	Give a cautious overall interpretation considering objectives, limitations, multiplicity of analyses, results from similar studies, and other relevant evidence.	18, 20
Generalisability	#21	Discuss the generalisability (external validity) of the study results	20

Other Information

Funding	#22	Give the source of funding and the role of the funders for the present study and, if applicable, for the original study on which the present article is based	21

None The STROBE checklist is distributed under the terms of the Creative Commons Attribution License CC-BY. This checklist can be completed online using https://www.goodreports.org/, a tool made by the EQUATOR Network in collaboration with Penelope.ai

For peer review only
